# DBeQ derivative targets vacuolar protein sorting 4 functions in cancer cells and suppresses tumor growth in mice

**DOI:** 10.1016/j.jpet.2025.103524

**Published:** 2025-02-28

**Authors:** Kevin A. Fundora, Yan Zhuang, Kouta Hamamoto, Guifang Wang, Longgui Chen, Tatsuya Hattori, Xinwen Liang, Lei Bao, Venugopal Vangala, Fang Tian, Yoshinori Takahashi, Hong-Gang Wang

**Affiliations:** 1 Division of Pediatric Hematology and Oncology, Department of Pediatrics, The Pennsylvania State University College of Medicine, Hershey, Pennsylvania; 2 Department of Biochemistry and Molecular Biology, The Pennsylvania State University College of Medicine, Hershey, Pennsylvania; 3 Department of Pharmacology, The Pennsylvania State University College of Medicine, Hershey, Pennsylvania

**Keywords:** VPS4, ESCRT, Autophagy, Lysosomal membrane integrity, Apoptosis

## Abstract

Vacuolar protein sorting 4 (VPS4) is an AAA-ATPase that catalyzes the endosomal sorting complex required for transport-III disassembly, mediating various cellular membrane-remodeling processes including endolysosomal membrane repair and autophagosome closure. Humans have 2 VPS4 paralogs, *VPS4A* and *VPS4B*, and the loss of either paralog has been identified in a significant proportion of cancers, rendering them dependent on the remaining paralog for survival. In this study, we explored VPS4 inhibition as an anticancer strategy by investigating the mechanisms of VPS4 inhibition-induced cell death and developing small-molecule compounds that target VPS4 functions. We found that genetic inhibition of VPS4 triggered both caspase-8 (CASP8)-dependent apoptosis and caspase-independent cell death in osteosarcoma cells. We synthesized approximately 100 derivatives of the VPS4 and related AAA-ATPase valosin-containing protein inhibitor DBeQ and screened for their inhibitory effects on VPS4 ATPase activity using the EnzChek phosphate assay and a high-content assay monitoring GFP-CHMP4B puncta formation. In cells, the lead compound 4-107 caused endolysosomal damage, disrupted subsequent membrane repair, inhibited autophagy, and led to the accumulation of the endosomal sorting complex required for transport on membranes. These effects were accompanied by the stabilization of CASP8 on autophagosomal membranes, leading to the induction of CASP8-mediated apoptosis. Notably, the CASP8-mediated cell death induced by 4-107 was further enhanced by the loss of either VPS4 paralog. Moreover, 4-107 exhibited antitumor activity in a syngeneic mouse model of neuroblastoma. Our findings provide an important step for targeting VPS4 in cancer and developing VPS4 inhibitors as a cancer treatment strategy.

**Significance Statement:**

*VPS4A* and *VPS4B*, paralogs of the AAA-ATPase VPS4, are critical for cancer cell survival. This study reports that 4-107, a DBeQ derivative, inhibits VPS4 ATPase activity, induces CASP8-mediated apoptosis, and suppresses tumor growth in mice. This study supports the further development of VPS4A/B inhibitors as a promising anticancer treatment strategy.

## 1 Introduction

The endosomal sorting complex required for transport (ESCRT) machinery is a highly conserved set of protein subcomplexes that together perform reverse-topology membrane scission. During membrane remodeling, upstream subcomplexes such as ESCRT-I, ESCRT-II, and ALG-2-interacting protein X family proteins are recruited to target membranes. These subunits then recruit the downstream component ESCRT-III, which in turn recruits vacuolar protein sorting 4 (VPS4), a member of the ATPases associated with diverse cellular activities (AAA) family. ESCRT-III forms membrane-constricting filaments that are dynamically shuffled and ultimately disassembled by VPS4 resulting in membrane scission (Remec Pavlin and Hurley, 2020). ESCRTs mediate a wide variety of cellular processes such as multivesicular body formation, virus budding, cytokinetic abscission, nuclear envelope maintenance, endolysosomal and plasma membrane repair, and autophagosome closure (Bohannon and Hanson, 2020).

The human genome encodes 2 VPS4 paralogs, *VPS4A* and *VPS4B*, which are located on chromosomes 16q and 18q, respectively, and are ubiquitously expressed (Scheuring et al, 2001; Beyer et al, 2003). The gene products share 80% protein sequence homology, which is reflected in their functional redundancy and ability to form heterohexamers for ESCRT disassembly (Scheuring et al, 2001; Beyer et al, 2003). Recent studies on somatic copy number alterations and cancer dependency genes have revealed that *VPS4A* or *VPS4B* frequently undergoes loss of heterozygosity (LOH) in various types of cancers and that more than 60% of cancer cells require the remaining paralog for their survival (McDonald et al, 2017; Neggers et al, 2020; Szymańska et al, 2020). Thus, targeting VPS4 in these cancers has emerged as an appealing treatment strategy to selectively induce synthetic lethality in cancer cells with *VPS4* LOH while exhibiting minimal toxicity against *VPS4*-intact noncancerous cells. However, the precise mechanisms of cell death induced by VPS4 inhibition remain to be clarified. ESCRT inhibition has been shown to induce proapoptotic signaling, leading to the induction of caspase-mediated apoptosis (Rodahl et al, 2009; Szymańska et al, 2020; Hattori et al, 2023). Mechanistically, we have recently found that autophagosomal membranes accumulated upon ESCRT inhibition serve as activation platforms for the intracellular death-inducing signaling complex (iDISC) to initiate caspase-8 (CASP8)-mediated apoptosis (Young et al, 2012; Hattori et al, 2021). Additionally, ESCRT inhibition can also induce caspase-independent, yet receptor-interacting serine/threonine kinase 1 (RIPK1)-dependent, cell death (Gong et al, 2017; Szymańska et al, 2020).

To date, 2 small molecule compounds that exhibit inhibitory activity against VPS4 have been reported. The ATP-competitive inhibitor DBeQ and allosteric inhibitor MSC1094308 were found to inhibit VPS4B ATPase activity in vitro with DBeQ also disrupting VPS4-mediated functions in yeast (Magnaghi et al, 2013; Zhang et al, 2016; Pöhler et al, 2018). However, these compounds are not specific to VPS4, as their main target is the related AAA-ATPase valosin-containing protein (VCP; Chou et al, 2011; Pöhler et al, 2018). VCP is vital for maintaining cell homeostasis by mediating the proteasomal degradation of misfolded proteins (Valimehr et al, 2023). Therefore, to harness the widespread vulnerability of cancers to VPS4 targeting, the development of potent and selective VPS4 inhibitors (VPS4i) is urgently needed, along with a better understanding of the mechanisms by which VPS4 inhibition triggers cell death. In this study, we found that inhibiting VPS4 via inducible expression of dominant-negative VPS4A or knockdown of *VPS4A* in *VPS4B*-deficient cells triggered both CASP8-dependent and caspase-independent cell death pathways. Moreover, our VPS4i-screening efforts based on optimization of DBeQ revealed lead compound 4-107 with improved potency and selectivity against VPS4 in cells. 4-107 disrupted several ESCRT-mediated pathways, including autophagy and endolysosomal membrane repair, leading to CASP8-mediated apoptosis with negligible effect on VCP-mediated pathways at inhibitory concentrations. Importantly, 4-107 induced cell death in a VPS4-sensitive manner and suppressed tumor growth in vivo. Our findings provide valuable insights for the future development of VPS4-targeted therapies for patients with cancers exhibiting *VPS4* LOH.

## 2 Materials and methods

### 2.1 Reagents

The following primary antibodies were used for immunoblotting (IB) and immunofluorescence (IF): mouse antibodies against ACTB/*β*-actin (Sigma-Aldrich; A5441; IB, 1:10,000), VPS4B (Santa Cruz Biotechnology; sc-377162; IB, 1:100), rabbit antibodies against ATF4 (Proteintech; 10835; IB, 1:1000), ATG5 (Cell Signaling Technology; 12994; IB, 1:1000), cleaved CASP3 (Cell Signaling Technology; 9661; IB, 1:1000), cleaved CASP8 (Cell Signaling Technology; 9496; IB, 1:1000), green fluorescent protein (GFP) (Cell Signaling Technology; 2956; IB, 1:1000), HSPA5 (Cell Signaling Technology; 3177; IB, 1:1000), K48-linkage specific polyubiquitin (Fisher Scientific; 50-190-885; IB, 1:2000), MAP1LC3B (LC3; Novus Biologicals; NB100-2220; IB, 1:4000; Cell Signaling Technology; 3868; IF, 1:200), PARP (Cell Signaling Technology; 9542; IB, 1:1000), pro-CASP8 (Abcam; ab108333; IB, 1:1000), VPS4A (Proteintech; 14272-1-AP; IB, 1:600), and guinea pig antibody against SQSTM1 (American Research Products; 03-GP62-C; IB, 1:5000; IF, 1:400). The following secondary antibodies were used for IB:HRP-conjugated goat antibody against rabbit IgG (Proteintech; SA00001-2; 1:2000), IRDye 680RD donkey antibodies against guinea pig IgG (LI-COR; 926-68077; 1:10,000), mouse IgG (LI-COR; 926-68072; 1:10,000), IRDye 800CW donkey antibody against rabbit IgG (LI-COR; 926-32213; 1:10,000), and IRDye 800CW goat antibody against rabbit IgG (LI-COR; 926-32211; 1:10,000). The following secondary antibodies were used for IF:Alexa Fluor 488-conjugated goat antibody against guinea pig IgG (Invitrogen; A11073; 1:1500), Alexa Fluor 700-conjugated goat antibody against rabbit IgG (Invitrogen; A11008; 1:1000). The following plasmids were obtained from Addgene:epiCRISPR (135960, Yongming Wang) (Xie et al, 2017), FUGW-PK-hLC3 (61460, Isei Tanida) (Tanida et al, 2014), lentiCas9-Blast (52962, Feng Zhang) (Sanjana et al, 2014), lentiCRISPR v2 (52961, Feng Zhang) (Sanjana et al, 2014), LRG (Lenti_sgRNA_EFS_GFP) (65656, Christopher Vakoc) (Shi et al, 2015), pEGFP-VPS4-E228Q (80351, Wesley Sundquist) (Votteler et al, 2016), pmCherry-Gal3 (85662, Hemmo Meyer) (Papadopoulos et al, 2016), pQE9-His-p97(wt) (14666, Graham Warren) (Meyer et al, 2000), TfR-pHuji (61505, David Perrais) (Shen et al, 2014). The following plasmids were gifted: pLNCX2-mEGFP-CHMP4B (Sanford Simon; The Rockefeller University; Bleck et al, 2014), and pET28a (Xuejun Jiang; Memorial Sloan Kettering Cancer Center). sgATG7-Cas9-2A-GFP (sc-400997) and sgCASP8-Cas9-2A-GFP (sc-400147) plasmid pools were purchased from Santa Cruz Biotechnology (Dallas, TX). For CRISPR-Cas9-mediated gene editing, sgRNAs listed in Supplemental Table 1 were subcloned into the BsmBI site of lentiCRISPR v2 or the SapI site of epiCRISPR. LRG plasmids encoding 3 different *VPS37A* sgRNAs were generated as previously described (Takahashi et al, 2019). To create inducible shRNA (i-shRNA) constructs, shRNAs listed in Supplemental Table 1 were subcloned into the BsbI site of pRSITEP-U6Tet-(shRNA)-EF1-TetRep-2A-Puro (Cellecta; SVSHU6TEP-L). pCDH-TRE3G-GFP-EF1*α*-Puro-tPT2A-Tet-On 3G, pCDH-TRE3G-GFP-VPS4A^E228Q^-EF1*α*-Puro-tPT2A-Tet-On 3G, pCDH1-CMV-MCS-SV40-Hygro, pCDH1-CMV-HT-LC3B-SV40-Hygro, pCDH1-CMV-GFP-VPS37A-EF1-Puro, pCDH1-myc-CASP8^C360A^-KN151-SV40-Hygro, and pCDH1-HA-CASP8^C360A^-LC151-SV40-Hygro were generated as previously described (Takahashi et al, 2019; Hattori et al, 2021; Hamamoto et al, 2024). To make pCDH-CMV-pHuji-LC3B-SV40-Hygro, pHuji from TfR-pHuji was subcloned into the NheI-XhoI site of pCDH1-CMV-HT-LC3B-SV40-Hygro. To generate pCDH-pHuji-LC3B-tPT2A-GFP-CHMP4B-SV40-Hygro, pHuji-LC3B from pCDH-CMV-pHuji-LC3B-SV40-Hygro, GFP-CHMP4B from pLNCX2-mEGFP-CHMP4B, and tPT2A were subcloned into the XbaI-BamHI site of pCDH1-CMV-MCS-SV40-Hygro using Gibson assembly. To make pCDH-UbC-mCherry-LGALS3-SV40-Bleo, mCherry-LGALS3 from pmCherry-Gal3 was subcloned into the NheI-EcoRI site of FUGW-PK-hLC3. To generate pET28a-VPS4B, VPS4B cDNA was subcloned into the BamHI-XhoI site of pET28a. Oligonucleotides used for plasmid construction are listed in Supplemental Table 1. All other reagents were obtained from the following sources: antibiotic antimycotic solution (AA; Cytiva; SV30079.01), ATP disodium salt hydrate (Sigma-Aldrich; A2383), bafilomycin A1 (BafA1; LC Laboratories; B-1080), Caspase-Glo 3/7 assay kit (VWR; G8091), Caspase-Glo 8 assay kit (VWR; G8201), CB-5083 (MedChemExpress; HY-12861), chloroquine diphosphate (CQ; Sigma-Aldrich; C6628), DBeQ (MedChemExpress; HY-15945), Dulbecco’s modified Eagle’s medium (high glucose) with sodium pyruvate, without amino acids (starvation media [SM]; Wako Chemicals; 048-33575), doxycycline hyclate (Dox; Sigma-Aldrich; D9891), DMEM (Corning; 10-013-CV), EnzChek Phosphate assay kit (Fisher Scientific; E6646), dimethyl sulfoxide (DMSO; Fisher Scientific; BP231), Hoechst 33342 solution (BD Biosciences; 561908), IGEPAL CA-630 (Sigma-Aldrich; 56741), jetPRIME transfection reagent (VWR; 89129-924), Matrigel Matrix (Fisher Scientific; CB-40234), McCoy’s 5A medium (Corning; 10-050-CV), normal goat serum (NGS; Sigma-Aldrich; G9023), paraformaldehyde (PFA; Electron Microscopy Sciences; 15710), phenylmethylsulfonyl fluoride (PMSF; Sigma-Aldrich; P7626), phosphatase inhibitor cocktail 2 (Sigma-Aldrich; P5726), phosphatase inhibitor cocktail 3 (Sigma-Aldrich; P0044), protease inhibitor cocktail (Sigma-Aldrich; P8340), Tet system-approved FBS (Thermo Scientific; A4736301), YOYO-3 iodide (Fisher Scientific; Y3606), z-IETD-fmk (Selleck Chemicals; S7314), and z-VAD-fmk (Selleck Chemicals; S7023).

### 2.2 Cell culture, transfection, and transduction

U2OS (HTB-96), 293T/17 (CRL-1128), and Phoenix-AMPHO cells (CRL-3213) were obtained from American Type Culture Collection. 9464D cells were a kind gift from Paul Sondel (University of Wisconsin). Cells were cultured in McCoy’s 5A medium (U2OS) or DMEM (293T/17, Phoenix-AMPHO, 9464D) supplemented with 1× AA and 10% FBS, or Tet system-approved FBS for i-shRNA-expressing cells, at 37 °C and 5% CO_2_. All cell lines used in this study were routinely tested for mycoplasma and were negative. Lentivirus- and retrovirus-mediated transduction was performed as previously described (Takahashi et al, 2019). CRISPR-Cas9-mediated gene knockout (cr-) U2OS cell lines, cr*ATG7*, cr*CASP8*, and cr*VPS37A*, were generated as previously described (Takahashi et al, 2019; Hattori et al, 2021). To obtain cr*ATG5* cells, parental cells were transduced with lentivirus encoding lentiCRISPR v2-sgATG5. cr*VPS4A* and cr*VPS4B* cells were generated via transient transfection of parental cells with epiCRISPR-sgRNA plasmids using jetPRIME transfection reagent. After selection and single clone isolation via limiting dilution, gene knockout was confirmed by immunoblotting. Single knockout clones (cr*ATG5*, *n* = 4; cr*VPS4A*, *n* = 5; cr*VPS4B*, *n* = 6) were pooled together and used for experiments.

### 2.3 Immunoblotting

Whole-cell lysates were prepared in radio-immunoprecipitation buffer (25 mM Tris-HCl, pH 8.0, 150 mM NaCl, 1 mM EDTA, 1% v/v IGEPAL CA-630, 0.5% w/v sodium deoxycholate, and 1% v/v SDS) containing protease (1:250) and phosphatase inhibitor cocktails (1:100) then resolved via SDS-PAGE and probed with the indicated antibodies. Signals were acquired with the Odyssey CLx (LI-COR) for fluorescence or Odyssey XF (LI-COR) for chemiluminescence detection and visualized in Image Studio version 6 (LI-COR).

### 2.4 Cell death assays

To measure caspase activity, cells cultured in white opaque-bottom 384-well plates were incubated in the presence or absence of 1 *μ*g/mL Dox for 24 hours. Caspase-Glo 8 or 3/7 reagent was then added 1:1 and allowed to incubate for 90 minutes at RT followed by luminescence measurement using the CLARIOstar microplate reader (BMG LABTECH). Signals had background luminescence subtracted and then normalized to the respective Dox-untreated i-GFP-expressing parental controls to obtain relative CASP8-like LETDase or CASP3/7-like DEVDase activities. For live-cell quantification, the cells seeded on black clear-bottom 96-well plates were incubated with the indicated compounds (0.1% to 0.3% DMSO final) in the presence of 100 nM YOYO-3, a cell-impermeant DNA-binding fluorescent probe that stains membrane integrity-impaired, dead cells. The dye has been successfully used to monitor the induction of CASP8-mediated cell death upon ESCRT inhibition (Hattori et al, 2021, 2023). For i-shRNA experiments, the media was replenished every 2 days. Phase and fluorescence images were obtained at the indicated intervals using the IncuCyte S3 Live-Cell Analysis System (Essen BioScience; 10× objective) and then analyzed using the Cell-by-Cell Analysis module (Essen BioScience; 9600-0031) of IncuCyte S3 Software (Essen BioScience; Version 2019B Rev2). To quantify the percentage of dead GFP-expressing cells, the GFP and YOYO-3 double-positive cell population was divided by the total GFP-positive cell population.

### 2.5 High-content microscopy

Live-cell fluorescence imaging and analysis were performed using the CellInsight CX7 LZR high-content screening platform with Onstage Incubator (Thermo Scientific; 37 °C, 5% CO_2_, 20% O_2_, 60% humidity). SM was acclimatized at 37 °C and 5% CO_2_ for at least 30 minutes prior to all experiments for pH equilibration. For cell-based VPS4i screen, U2OS cells stably expressing N-terminal GFP-tagged charged multivesicular body protein 4B (GFP-CHMP4B) were cultured in black clear-bottom 96-well plates (Corning; 3603) at a density of 6000 cells/well. Cells were stained with Hoechst 33342 (1:1000) for 15 minutes at 37 °C, rinsed with 1× Dulbecco's phosphate-buffered saline (DPBS), and treated with SM containing 0.1% DMSO or 1 *μ*M test compounds. Plates were then scanned using the 20× objective for 1 hour with ≥100 cells/well across 6 fields/well. Fluorescence intensity of GFP-CHMP4B foci per cell for each compound was normalized to that of 4-107. For all other assays, the cells were cultured overnight in black glass-bottom 96-well plates (Cellvis; P96-1.5H-N) then stained, rinsed, and treated with SM containing the indicated compounds (0.1% DMSO or 0.1% DMSO/0.1% ethanol [vehicle] final). Plates were scanned using the 20× NA .70 objective at the indicated intervals with ≥ 300 cells/well across 3 fields/well.

### 2.6 Recombinant protein expression and purification

*Escherichia coli* BL21(DE3) cells were transformed with pET28a-VPS4B and pQE9-His-p97(wt) (VCP) and grown in 25 mL LB medium at 37 °C overnight. The cells were then centrifuged and transferred to a 500 mL fresh LB medium. When OD_600_ = 0.6–0.9, 1 mM isopropyl *β*-D-1-thiogalactopyranoside was added to induce protein expression and cells were grown at 20 °C for 4 or 16 hours for VCP or VPS4B, respectively. The cells were harvested by centrifugation and stored at −80 °C until use. VPS4B was purified as previously described (Cupido et al, 2019) using the following buffers: Buffer A1 (20 mM HEPES-NaOH, pH 7.6, 500 mM NaCl, 2 mM MgCl_2_, 10% v/v glycerol, 0.5 mM dithiothreitol (DTT), 1 mM PMSF, 1 mM ATP, complete EDTA-free protease inhibitor cocktail), Buffer A2 (20 mM HEPES-NaOH, pH 7.6, 500 mM NaCl, 2 mM MgCl_2_, 0.5 mM DTT, 1 mM PMSF, 1 mM ATP, 15 mM imidazole), Buffer A3 (20 mM HEPES-NaOH, pH 7.6, 500 mM NaCl, 2 mM MgCl_2_, 0.5 mM DTT, 1 mM PMSF, 1 mM ATP, 400 mM imidazole), Buffer A4 (25 mM HEPES-NaOH, pH 7.6, 2 mM MgCl_2_, 1 mM DTT), Buffer A5 (25 mM HEPES-NaOH, pH 7.6, 1 M NaCl, 2 mM MgCl_2_, 1 mM DTT), Buffer A6 (50 mM HEPES-NaOH, pH 7.6, 150 mM NaCl, 3 mM MgCl_2_, 2 mM DTT). VCP was purified based on literature precedents (Meyer et al, 2000; Dalal et al, 2004) using the following buffers: Buffer B1 (50 mM HEPES, pH 7.6, 500 mM NaCl, 5% v/v glycerol, 20 mM imidazole, 2 mM β-mercaptoethanol (BME), complete EDTA-free protease inhibitor cocktail), Buffer B2 (50 mM HEPES, pH 7.6, 500 mM NaCl, 5% v/v glycerol, 500 mM imidazole, 2 mM BME), and Buffer B3 (50 mM HEPES-KOH, pH 7.4, 150 mM NaCl, 5% v/v glycerol, 0.5 mM ATP, 1 mM BME). Cell pellets containing expressed protein were suspended in Buffer A1/B1 and then incubated with 2.5 mg of lysozyme for 30 minutes on ice. Suspended cells were disrupted by M-110P microfluidizer, and cell debris was removed by centrifugation. Supernatants were collected and loaded onto a Ni-NTA column (GE Healthcare). For VPS4B, the column was washed with Buffer A2 and eluted with Buffer A3. For VCP, the column was extensively washed with Buffer B1 and eluted with Buffer B2. Eluted VPS4B was diluted with 4 volumes of Buffer A4 and loaded onto a MonoQ column (GE Healthcare) equilibrated in Buffer A4, then fractionated over a gradient with Buffer A5. Combined MonoQ VPS4B fractions and eluted VCP were then concentrated and purified on a Superdex 75 column (GE Healthcare) in Buffer A6 or Buffer B3, respectively.

### 2.7 ATPase assays

VPS4B and VCP ATPase activities were measured using the EnzChek Phosphate assay for the quantification of released phosphate. Assay buffers were based on literature precedents (Cupido et al, 2019, 2021) as follows: VPS4B buffer (20 mM HEPES-KOH, pH 7.4, 25 mM KOAc, 2 mM MgCl_2_, 5 mM (NH_4_)_2_SO_4_, 0.5 mM CaCl_2_, 2.5 mM DTT, 0.1 mg/mL BSA, 0.005% v/v Triton X-100), VCP buffer (20 mM HEPES-NaOH, pH 7.4, 20 mM NaCl, 10 mM MgCl_2_, 5 mM (NH_4_)_2_SO_4_, 0.5 mM CaCl_2_, 2.5 mM DTT, 0.1 mg/mL BSA, 0.005% v/v Triton X-100). In black clear-bottom 384-well plates, 22.5 *μ*L of 5% DMSO or 40× test compounds diluted in the respective assay buffer was added to each well. ATPases were diluted to 1 *μ*M in the respective buffers containing 2× EnzChek Phosphate assay coupling reagents of 400 *μ*M MESG and 2 U of PNP, then 25 *μ*L was added to each well. Plates were mixed at 1000 rpm for 30 minutes at RT while being protected from light. The reaction was initiated by adding 2.5 *μ*L of 20 mM MgATP or water followed by mixing at 1200 rpm for 30 seconds at RT. Plates were measured at 360 nm using a CLARIOstar plate reader (BMG LABTECH) for 20 minutes at 37 °C. ATPase activity was calculated as the amount of phosphate released over a 5-minute interval under linear initial velocity conditions. The remaining ATPase activity was calculated by normalizing compound-treated activity values to the DMSO control. IC_50_ was calculated by fitting normalized values to a sigmoidal dose-response curve with Hill slope = −1 using Graph Pad Prism 10.0.

### 2.8 Cellular thermal shift assay (CETSA)

The VPS4B CETSA was performed as previously described with modification (Jafari et al, 2014). In 10-cm dishes, near-confluent U2OS cells were rinsed with 1× DPBS and treated with the indicated compounds in acclimatized SM for 1 hour at 37 °C (0.1% DMSO). Cells were trypsinized, pelleted at 300 × *g* for 5 minutes, and washed with 1× PBS. After repeat pelleting at 300 × *g* for 5 minutes, the cells were resuspended in 1× PBS with protease inhibitor cocktail (1:250) and aliquoted into PCR strips with 20 *μ*L per tube. PCR strips for each treatment were heated on a temperature gradient for 3 minutes using a thermal cycler (Bio-Rad Laboratories), followed by incubation at RT for 3 minutes. Cells were mixed with IGEPAL CA-630 (0.25% v/v final) and then lysed using 3 freeze-thaw cycles of 30 seconds in liquid nitrogen, 30 seconds in RT water bath, and 10 seconds vortex mixing. Cell lysates were transferred to 1.5 mL tubes, centrifuged at 20,000 × *g* for 20 minutes at 4 °C, and the supernatant containing soluble protein was extracted. To normalize protein loading for SDS-PAGE, the soluble protein concentration of each 37 °C sample was quantified via the Pierce BCA assay (Promega), and a volume equal to 10 *μ*g of protein was used for all samples from the same treatment. The samples were then resolved via SDS-PAGE and subjected to immunoblotting. Signals were acquired with the Odyssey CLx (LI-COR) and quantified via densitometry using Image Studio version 6 (LI-COR). The remaining VPS4B levels were calculated by first obtaining the ratio of VPS4B:ACTB band intensities of each sample and then normalizing these ratios to that of the respective 37 °C sample. The temperature where 50% of the soluble VPS4B aggregated (VPS4B *T*_agg_) was calculated by fitting normalized values to a sigmoidal temperature-response curve with variable slope using Graph Pad Prism 10.0.

### 2.9 Correlative light-electron microscopy

cr*VPS37A* U2OS cells stably expressing GFP-VPS37A and pHuji-LC3 were grown overnight on a gridded glass-bottom dish (MatTek; P35G-1.5-14-C-GRD), starved in the presence of 1 *μ*M 4-107 (0.1% DMSO) for 1 hour, and fixed in 4% PFA/0.1% glutaraldehyde-containing DPBS for 20 minutes at RT. Cells of interest were identified by correlating the grid and 3-dimensional images were obtained using a Leica AOBS SP8 laser-scanning confocal microscope (63× water or oil-immersion lens), deconvolved using Huygens deconvolution software (Scientific Volume Imaging), and analyzed using Imaris software (Bitplane). Cells were then incubated in 2% PFA/2.5% glutaraldehyde in 0.1 M phosphate buffer, pH 7.4, for 1 hour at RT, followed by 1% osmium tetroxide/1.5% potassium ferrocyanide in 0.1 M phosphate buffer, pH 7.4, for 30 minutes at RT. Cells were then dehydrated in a graduated ethanol series, followed by acetone, and embedded in LX-112 (Ladd Research). Thin sections (65 nm) were stained with uranyl acetate and lead citrate and analyzed using a JEOL JEM1400 Transmission Electron Microscope (JEOL USA).

### 2.10 Bimolecular fluorescence complementation assay

Cells were co-transduced with lentivirus encoding myc-CASP8^C360A^-KN151 and HA-CASP8^C360A^-LC151 and seeded on Lab-Tek II Chamber Slides (Thermo Fisher Scientific; 154941). Ninety-six hours after transduction, the cells were starved in the presence of 1 *μ*M 4-107 (0.1% DMSO) for 2 hours, then fixed in 4% PFA for 7 minutes, washed 3 times with 1× DPBS, and permeabilized in methanol at −20 °C for 5 minutes. Cells were then blocked in 10% NGS for 1 hour at RT followed by primary antibody incubation in 1% NGS overnight at 4 °C and secondary antibody incubation in 1% NGS for 1 hour at RT. Leica AOBS SP8 laser scanning confocal microscope (oil-immersion [1.2 numerical aperture] lens) with highly sensitive HyD detectors and Leica image acquisition software LAX were used to obtain fluorescence images. Acquired images were deconvolved using Huygens deconvolution software (Scientific Volume Imaging), and analyzed using Imaris software (Bitplane) and Volocity software (PerkinElmer) without gamma adjustment. PBS was used as the imaging medium.

### 2.11 In vitro ADME studies of 4-107

All in vitro absorption, distribution, metabolism, and excretion (ADME) studies were conducted by BioDuro-Sundia. Thermodynamic solubility was determined by mixing solid 4-107 with FaSSIF-V2 buffer, pH 6.5, for 16 hours at RT under agitation then subjecting the sample to liquid chromatography-mass spectrometry/mass spectrometry analysis. Caco-2 permeability, human liver microsomal stability, and human plasma protein binding assays as well as liquid chromatography-mass spectrometry/mass spectrometry analyses were performed following standard protocols previously described in detail (Kazakova et al, 2023).

### 2.12 In vivo toxicity studies of 4-107

All animal studies were performed in accordance with the guidelines of the Institutional Animal Care and Use Committee at the Penn State College of Medicine (IACUC # PRAMS201145989). To determine the maximum tolerated dose (MTD) of 4-107, escalating and acute dose studies were performed. The escalating MTD study used an intra-animal design where each mouse received daily doses of 4-107 that increased 50% each day from a starting dose of 5 mg/kg. Six female C57BL/6J (B6) (The Jackson Laboratory; 000664) were administered 4-107 either orally via oral gavage or via intraperitoneal injection with 3 mice per group. The acute MTD study had groups of mice receive daily consistent doses of 4-107 i.p. Here, B6 mice were administered 4-107 at either 5 (*n* = 4; 2 males [M], 2 females [F]), 10 (*n* = 4; 2 M, 2 F), 15 (*n* = 5; all M), or 20 mg/kg (*n* = 5; all M). In both studies, 4-107 dissolved in DMSO was mixed into 30% propylene glycol, 5% Tween 80, and 65% of 5% dextrose in water (PTD [vehicle]) with a final DMSO concentration of ≤ 3%. Dosing continued until mice met the toxicity endpoints of > 15% weight loss or a body condition score (BCS) < 2, at which point they were euthanized. BCS was determined by posture and prominence of skeletal features upon visual inspection and palpation, where BCS < 2 indicated a hunched posture and emaciation. Mice with > 15% weight loss were considered BCS < 2. The MTD was determined as the highest dose of 4-107 administered without mice reaching the toxicity endpoints.

### 2.13 In vivo tumor studies of 4-107

To establish a syngeneic tumor mouse model, 9464D cells were resuspended in 50% Matrigel Matrix in 1× PBS at a concentration of 2 × 10^7^ cells/mL and a total of 0.1 mL of this mixture (1 × 10^6^ cells) was subcutaneously injected into the flank of each of the 6 to 8-week-old B6 mice (*n* = 19; 12 M, 7 F). Fourteen days after engraftment, mice began daily intraperitoneal administration of either vehicle (*n* = 9; 6 M, 3 F) or 5 mg/kg 4-107 (*n* = 10; 6 M, 4 F). Mouse tumor volume was measured weekly, then every 1–3 days once vehicle-treated tumors reached 1000–2000 mm^3^. Tumor volume was calculated as *πLW*^2^/6 using length (*L*) and width (*W*) caliper measurements. Mouse body weight was measured daily. Humane endpoints were defined as tumor volume reaching 4000 mm^3^ or mice reaching the aforementioned toxicity endpoints, at which point the affected mice were euthanized. The experimental endpoint was determined as the time when the majority of tumors from the control group reached 4000 mm^3^.

### 2.14 Chemistry

The proton nuclear magnetic resonance (^1^H NMR) spectra were determined using an AVANCE II (500 MHz) spectrometer (Bruker). Chemical shifts for ^1^H NMR are reported in parts per million (ppm) downfield from tetramethylsilane (*δ*) as an internal standard in deuterated solvent and coupling constants (*J*) are given in Hertz (Hz). The following abbreviations are used for spin multiplicity: s = singlet, d = doublet, t = triplet, q = quartet, dd = doublet of doublet, dt = doublet of triplet, m = multiplet, and br s = broad singlet. Reaction progress was monitored by thin-layer chromatography (TLC) analysis on silica gel 60 F_254_ plates (Sigma-Aldrich; 60778) or an NH TLC plate (Fuji Silysia Chemical). Column chromatography was carried out on a silica gel column (Chromatorex NH-DM1020, 100–200 mesh; Fuji Silysia Chemical). Low-resolution mass spectra (MS) were acquired using an Agilent Waters Alliance ACQUITY QDa. The column used was a Waters X-Bridge C18 (4.6 × 50 mm I.D., 3.5 *μ*m) with a temperature of 40 °C and a flow rate of 0.80 mL/min. Mobile phase: Condition 1: Mobile phases A and B under acidic conditions were 0.1% HCOOH in water and 0.1% HCOOH in MeCN, respectively. The proportion of mobile phase B was increased linearly from 5% to 90% over 0.9 minutes, and 90% over the next 1.1 minutes; or from 5 to 100% over 1.6 minutes, and 100% over the next 1.4 minutes; or from 5 to 100% over 3.0 minutes, and 100% over the next 1.0 minutes. Condition 2: Mobile phases A and B under neutral conditions were a mixture of 5 mmol/L AcONH_4_ and MeCN (9:1, v/v) and a mixture of 5 mmol/L AcONH_4_ and MeCN (1:9, v/v), respectively. The proportion of mobile phase B was increased linearly from 5% to 90% over 0.9 minutes, and 90% over the next 1.1 minutes. All commercially available solvents and reagents were used without further purification. Yields were not optimized. Abbreviations are used as follows: AcOH, acetic acid; DMSO, dimethyl sulfoxide; DMSO-d_6_, deuterated dimethyl sulfoxide; Et_3_N, triethylamine; EtOAc, ethyl acetate; EtOH, ethanol; MeCN, acetonitrile; MeOH, methanol; Pd/C, palladium on carbon; THF, tetrahydrofuran.

### 2.15 Representative compound synthesis: N^2^,N^4^-dibenzyl-8-methoxyquinazoline-2,4-diamine (4, compound 4-107)

**Scheme 1:**
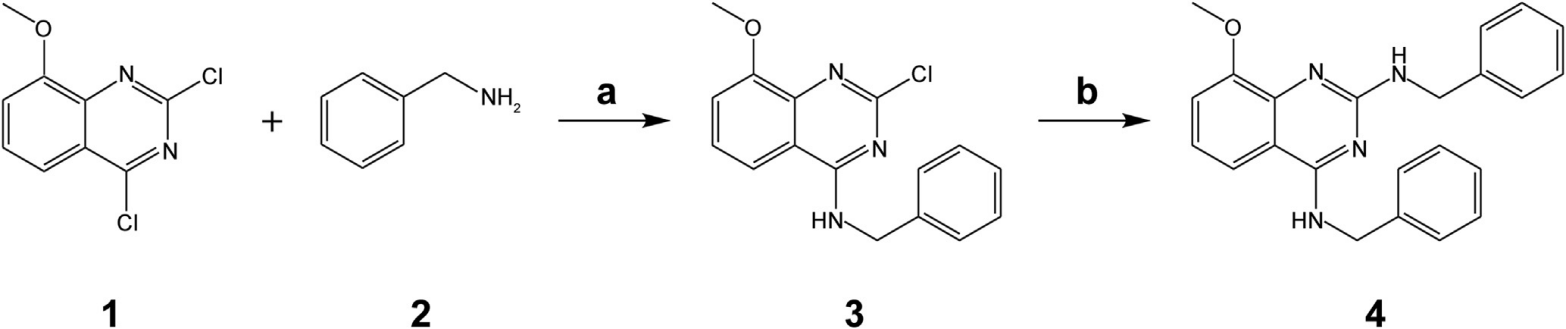
General synthetic route of compound 4 (4-107). (a) NH_4_OH, MeCN, RT 16 hours; (b) 2, Pd(OAc)_2_, Cs_2_CO_3_, C98327-87-8, dioxane, 140 °C, 12 hours.

The overall synthetic route is described in Scheme 1. Briefly, to a suspension of 2,4-dichloro-8-methoxyquinazoline (1, 460 mg, 2 mmol) in MeCN (10 mL) was added benzylamine (2, 0.24 mL, 2.20 mmol, 1.1 equiv.), NH_4_OH 1 mL. The mixture was stirred at RT overnight. The mixture was diluted with EtOAc, filtered, and the filtrate was concentrated to give intermediate 3 (white powder 0.5 g, 83%). To a RT solution of the intermediates (3) (350 mg, 1.17 mmol) and (2) (0.25 mL, 2.34 mmol) in 1,4-dioxane (10 mL) was added Cs_2_CO_3_ (456 mg, 1.4 mmol). The mixture was degassed and filled with nitrogen 3 times. Pd(OAc)_2_ (27 mg, 0.12 mmol) and C98327-87-8 (73 mg, 0.12 mmol) then were added. The resulting mixture was stirred at 140 °C for 12 hours and cooled to RT. The volatiles were evaporated in vacuo and the resulting residue was dissolved in methylene dichloride (50 mL), washed with water (20 mL) and brine (30 mL × 2), dried over Na_2_SO_4_, filtered, and evaporated in vacuo. The residue was purified by column chromatography (silica gel, hexane, EtOAc) to give the desired final product as a white solid (4, 4-107; 150 mg, yield: 35%, purity: >95%). ^1^H NMR (500 MHz, DMSO-d_6_) *δ* 8.311 (s, br. 1H), 7.593–7.577 (d, *J* = 8 Hz, 1H), 7.317–7.159 (m, 11H), 7.019–7.004 (d, *J* = 7.5 Hz, 1H), 6.969–6.937 (t, J = 8 Hz, 1H), 4.69 (s, 2H), 4.51 (s, 2H), 3.81 (s, 3H). ^13^C NMR (500 MHz, DMSO-d_6_) *δ* 160.4, 159.4, 153.3, 141.2, 140.5, 128.6, 128.5, 127.8, 127.7, 127.0, 126.7, 119.9, 114.7, 55.9, 44.5, 44.7. Low-resolution mass spectrometry (m/z): Calcd for Chemical Formula: C_23_H_22_N_4_O (M^+^) 370.18; found 371.3(M^+^H).

### 2.16 Statistical analyses

Statistical analyses were performed using unpaired two-tailed *t* test (2 groups) or one-way ANOVA followed by Tukey’s multiple comparison test (>2 groups) in Graph Pad Prism 10.0. The threshold for statistical significance for each test was set at 95% confidence (*P* ≤ .05).

## 3 Results

### 3.1 VPS4 genetic inhibition induces CASP8-dependent apoptosis and caspase-independent cell death

**Fig. 1:**
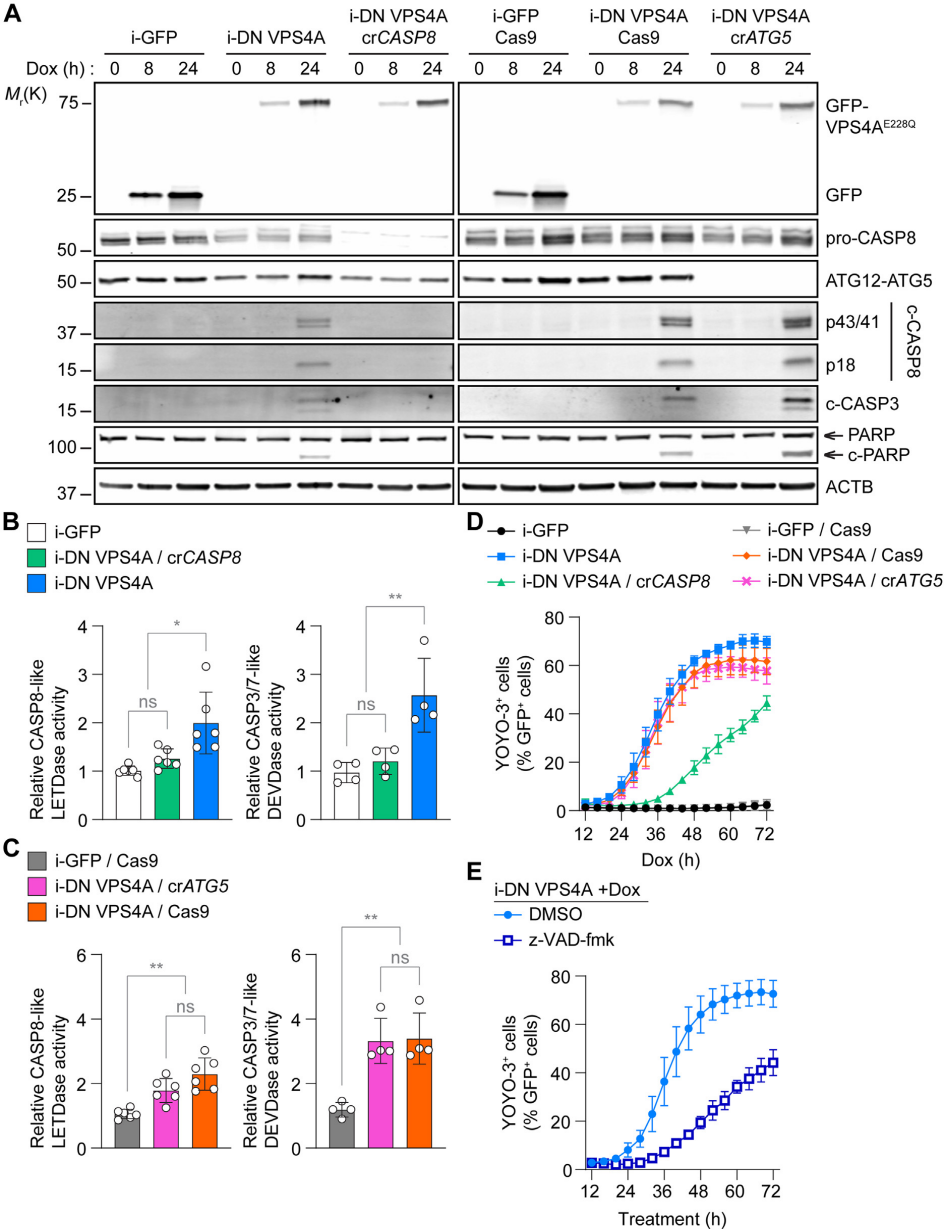
Dominant-negative (DN) VPS4A expression induces CASP8-dependent apoptosis and caspase-independent cell death in U2OS cells. (A) Immunoblot analysis of i-GFP- and i-DN VPS4A-expressing U2OS cells incubated in the presence or absence of 1 *μ*g/mL Dox for the indicated times. Representative blot shown from 3 independent experiments. (B and C) CASP8-like LETDase and CASP3/7-like DEVDase activities of i-GFP- and i-DN VPS4A-expressing cells incubated with 1 *μ*g/mL Dox for 24 hours. Each caspase-like activity was normalized to the respective value of Dox-untreated i-GFP-expressing parental cells. Data shown in B and C are from 4 (CASP3-like DEVDase) and 6 (CASP8-like LETDase) independent experiments, each with technical triplicates. (D) Live-cell quantification of GFP and YOYO-3 double-positive dead cells relative to the total GFP-positive population of i-GFP- and i-DN VPS4A-expressing cells incubated with 1 *μ*g/mL Dox. Data shown are from 3 independent experiments, each with technical triplicates. (E) Live-cell quantification of GFP and YOYO-3 double-positive dead cells relative to the total GFP-positive population of i-DN VPS4A-expressing cells incubated with 1 *μ*g/mL Dox in the presence or absence of 20 *μ*M z-VAD-fmk. Data shown are from 3 independent experiments, each with technical triplicates. All values in the graphs are mean ± SD, calculated using the mean values from each of the independent experiments. Statistical analysis was performed using one-way ANOVA followed by Tukey’s multiple comparison test; ns not significant, ∗*P* ≤ .05; ∗∗*P* < .01.

To investigate the mechanism of cell death induced by VPS4 inhibition, we established a doxycycline (Dox)-inducible system of N-terminal GFP-tagged, ATPase-deficient dominant-negative VPS4A^E228Q^ (i-DN VPS4A) and control GFP alone (i-GFP) in U2OS cells. For evaluating whether VPS4 inhibition leads to iDISC-mediated apoptosis, the inducible system was also stably expressed in *CASP8*- (cr*CASP8*) and *ATG5*-deficient (c*rATG5*) cells in which the initiator CASP8 and its localization to autophagosomal membranes for proximity induced-activation are disrupted, respectively. Immunoblot analysis showed that i-DN VPS4A expression was detectable within 8 hours of Dox treatment and induced apoptosis by 24 hours based on the cleavage of CASP8, CASP3, and PARP (Fig. 1A). In the absence of *CASP8*, neither CASP3 nor PARP cleavage was observed, indicating the role of CASP8 in initiating the caspase cascade upon VPS4 inhibition. Meanwhile, *ATG5* loss failed to inhibit apoptosis despite its critical role in iDISC assembly (Young et al, 2012). These trends in apoptosis were further reflected in caspase activities after 24 hours of i-DN VPS4A expression (Fig. 1, B and C). *CASP8*-deficient i-DN VPS4A-expressing cells showed similar CASP3/7 activity as i-GFP-expressing cells supporting the requirement of CASP8 in VSP4 inhibition-induced apoptosis. To further assess the role of CASP8 in VPS4 inhibition-induced cell death, we next monitored cell viability over time, based on the loss of plasma membrane integrity using YOYO-3 iodide. Consistently, we observed that the loss of *CASP8*, but not *ATG5*, protected against cell death from i-DN VPS4A expression (Fig. 1D). Despite VPS4 inhibition activating CASP8-dependent apoptosis, cell death was only partially rescued in cr*CASP8* cells. A similar response was observed when cells were treated with the pan-caspase inhibitor z-VAD-fmk (Fig. 1E).

**Fig. 2:**
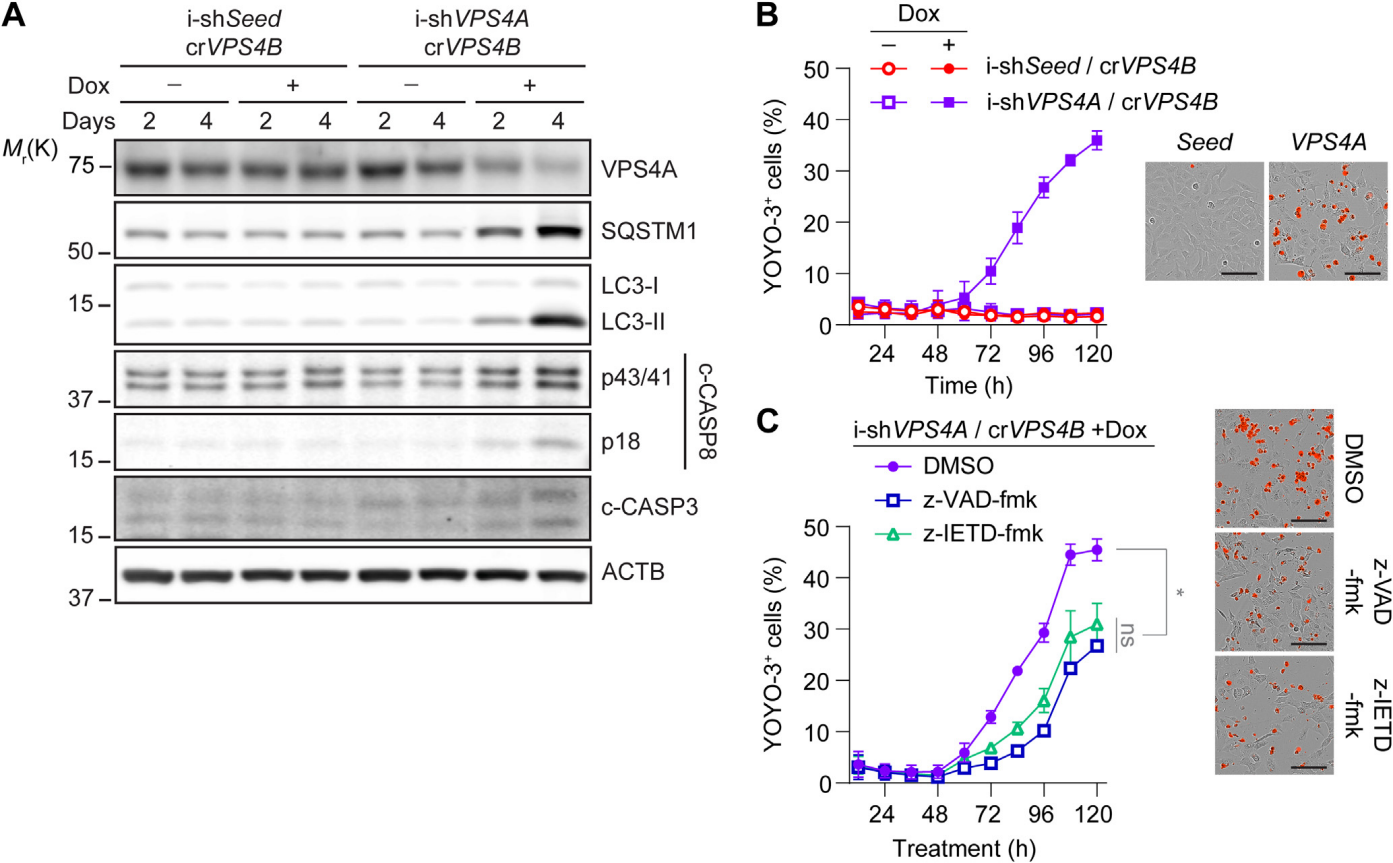
*VPS4A* depletion induces synthetic lethality in *VPS4B*-deficient cells in a CASP8-dependent and -independent manner. (A) Immunoblot analysis of i-sh*Seed*- and i-sh*VPS4A*-expressing cr*VPS4B* U2OS cells incubated in the presence or absence of 1 *μ*g/mL Dox for 2 and 4 days. Representative blot shown from 4 independent experiments. (B) Live-cell quantification of YOYO-3-positive dead i-shRNA-expressing cells incubated in the presence or absence of 1 *μ*g/mL Dox. Data shown from 3 independent experiments, each with technical triplicates. (C) Live-cell quantification of YOYO-3-positive dead i-sh*VPS4A*-expressing cells incubated with 1 *μ*g/mL Dox in the presence or absence of 20 *μ*M z-VAD-fmk and 50 *μ*M z-IETD-fmk. Data shown are from 2 independent experiments, each with technical triplicates. Representative images of cells at the experimental endpoint are shown for B and C. Scale bars: 100 *μ*m. All values in the graphs are mean ± SD, calculated using the mean values from each of the independent experiments. Statistical analysis was performed using one-way ANOVA followed by Tukey’s multiple comparison test; ns not significant, ∗*P* ≤ .05.

To corroborate these findings and rule out potential artifacts from overexpression of DN VPS4A, we generated an additional model of VPS4 genetic inhibition by stably expressing Dox-inducible shRNA targeting either *VPS4A* (i-sh*VPS4A*) or a control sequence (i-sh*Seed*) in *VPS4B*-deficient (cr*VPS4B*) U2OS cells. Immunoblot analysis confirmed a Dox-specific and time-dependent depletion of *VPS4A* over 2–4 days that resulted in the inhibition of autophagy, as indicated by the increase in sequestosome 1 (SQSTM1) and membrane-conjugated microtubule-associated protein 1 light chain 3 (LC3-II), as well as the induction of apoptosis, as evidenced by the detection of the cleaved, active forms of CASP8 and CASP3 (Fig. 2A). Moreover, inducible depletion of *VPS4A* in cr*VPS4B* cells resulted in cell death after 48 hours of Dox treatment (Fig. 2B). Comparable with our findings with i-DN VPS4A expression, z-VAD-fmk, and CASP8 inhibitor z-IETD-fmk both partially rescued VPS4 synthetic cell death to the same extent (Fig. 2C). Collectively, these results indicate that VPS4 genetic inhibition induces CASP8-dependent apoptosis and caspase-independent cell death.

### 3.2 Establishment of VPS4 inhibitor screening assays

**Fig. 3:**
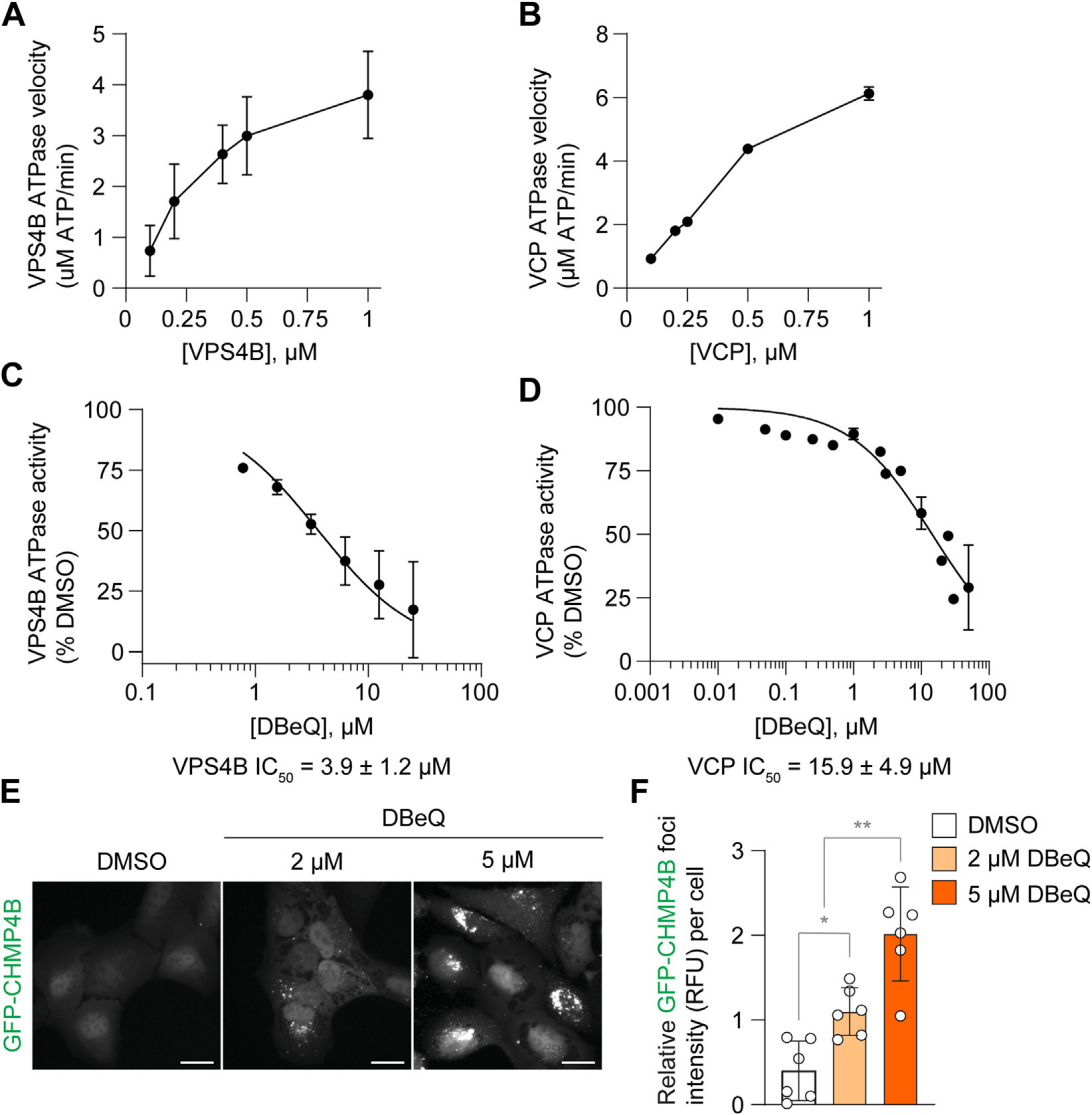
Establishment of enzyme- and cell-based assays for VPS4 inhibitor screening. (A and B) Initial ATPase velocities of recombinant human VPS4B (A) and VCP (B) at the indicated concentrations. Data shown are from 3 (A) or 2 (B) independent experiments, each with technical triplicates. (C and D) Remaining ATPase activities of VPS4B (C) and VCP (D) relative to DMSO when incubated with the indicated concentrations of DBeQ. Data shown are from 3 (C) or 2 (D) independent experiments, each with technical triplicates. (E) Representative high-content microscopy (HCM) images of GFP-CHMP4B-expressing U2OS cells starved in the presence or absence of 2 and 5 *μ*M DBeQ for 2 hours. Scale bars: 20 *μ*m. (F) HCM quantification of relative fluorescence intensity of GFP-CHMP4B foci per cell from E. Data (mean ± SD) shown are representative of 2 independent experiments, each with 6 technical replicates. For A-D, values in the graphs are mean ± SD, calculated using the mean values from each of the independent experiments. Statistical analysis was performed using one-way ANOVA followed by Tukey’s multiple comparison test; ns not significant, ∗*P* ≤ .05; ∗∗*P* < .01.

To identify VPS4i, we developed enzyme- and cell-based VPS4 functional assays to serve as VPS4i-screening platforms. For the enzyme-based assay, we used the colorimetric EnzChek Phosphate assay that detects free phosphate with the advantage of continuous measurement of ATP hydrolysis over time. Our purified recombinant human VPS4B construct exhibited concentration-dependent basal ATPase activity, and we chose 0.5 *μ*M for compound testing (Fig. 3A). Due to the high sequence and structural similarity of AAA-ATPases, we also established a VCP ATPase assay to evaluate hit selectivity. Our purified recombinant human VCP displayed a concentration-dependent increase in ATPase activity, and we ultimately chose 0.5 *μ*M for screening (Fig. 3B). To validate these assays, we tested DBeQ and observed dose-dependent inhibition of both VPS4B and VCP ATPase activities with IC_50_ values of 3.9 ± 1.2 *μ*M and 15.9 ± 4.9 *μ*M, respectively (Fig. 3, C and D). For the cell-based assay, our approach was based on how VPS4 inhibition causes the accumulation of ESCRT-III (Babst et al, 1998; Katoh et al, 2003). We used an N-terminal GFP-tagged CHMP4B reporter system because it is the most abundant ESCRT-III subunit (Katoh et al, 2004; Teis et al, 2008). Our aim was to test compounds under short-term serum and amino acid starvation to stimulate ESCRT activity for easier detection of inhibitors (Takahashi et al, 2018, 2019). We validated our cell-based assay using DBeQ and observed dose-dependent accumulation of GFP-CHMP4B in starved cells, consistent with VPS4 inhibition (Fig. 3, E and F). Overall, we established 2 systems for VPS4i screening, a VPS4B ATPase activity assay and a GFP-CHMP4B accumulation cell-based assay.

### 3.3 Structure-activity relationship of DBeQ for VPS4 inhibition

To optimize DBeQ as VPS4i, we initiated a structure-activity relationship (SAR) study of DBeQ using the aforementioned screening assays. Our approach explored substitutions on the quinazoline core (R^1^), 2-position (R^2^), and 4-amino positions (R^3^) (Table 1). Early into our efforts, an initial DBeQ analog with a methoxy group at the 8-position of the quinazoline core demonstrated similar ATPase inhibition along with a dramatic improvement in accumulating GFP-CHMP4B (Table 1, compound **1**). Changing the 8-position group to methyl, halo, and nitro substituents all reduced activity in both the enzyme- and cell-based assays (Table 1, compounds **2**–**5**) emphasizing that the electron-releasing 8-methoxy group was the preferred substitution. Attempts to change the position of the methoxy group resulted in analogs with comparable ATPase inhibition but diminished activity in cells (Table 1, compounds **6** and **7**). Given these findings, we decided to move forward with lead optimization of **1**, hereafter referred to as 4-107, by changing groups at the 2-position and 4-amino positions. At the 2-position, replacing the aminobenzyl group with polyaromatic heterocycles abolished activity in both assays (eg, compounds **8** and **9**, Table 1; Supplemental Table 2) and suggested the importance of maintaining the original moiety in 4-107. To investigate this, we incrementally modified this group by first holding the amino portion constant and replaced the benzyl moiety with 2- and 3-membered polyaromatic substituents that moderately reduced ATPase inhibition but ablated in-cell activity (eg, compounds **10** and **11**, Table 1; Supplemental Table 2). We next kept the aminomethyl moiety and added naphthyl groups, resulting in the first analogs with improved activity in the enzymatic assay yet were still weak in cells (Table 1, compounds **12** and **13**). A pattern was noted where the greater the deviation from the aminobenzyl group, the greater the reduction in cellular activity. Therefore, we decided to keep the original moiety and instead incorporate substituents onto the benzyl group. Analogs with ester, trifluoromethyl, and trifluoromethoxy substituents had notable activity in the enzyme assay (eg, compounds **14**–**16**, Table 1; Supplemental Table 2), but those with cyano, methyl, methoxy, and fluoro substituents were active in both assays (eg, compounds **17**–**20**, Table 1; Supplemental Table 2). Derivatives **19** and **20** were the first compounds to induce GFP-CHMP4B accumulation within the range of the lead 4-107. Meanwhile, modifications of the 4-amino position, including analogs of **19** and **20** with the same substituent on both benzyl moieties, failed to yield superior compounds (Table 1, compounds **21** and **22**, respectively). Here, we decided to move forward to evaluate the VPS4B-VCP selectivity of 4-107 and promising hits **17**–**20**. All compounds demonstrated selectivity for VPS4B over VCP with analogs **17**–**20** being more selective than 4-107 (Table 2). Ultimately, we elected the lead compound 4-107 for further functional characterization in this study due to its superiority in accumulating GFP-CHMP4B.

**Table 1:** Selected SAR of DBeQ analogs


Compound	R^1^	R^2^	R^3^	VPS4B ATPase (% DMSO)^a^	GFP-CHMP4B (% 4-107)^b^
DBeQ	5,6,7,8-H	NHBn	Bn	41.96 ± 7.53	8.93 ± 3.51
**1** (4-107)	8-OMe	NHBn	Bn	36.67 ± 1.73	100.00 ± 17.20
**2**	8-Me	NHBn	Bn	55.31 ± 5.04	6.69 ± 3.07
**3**	8-Cl	NHBn	Bn	71.88 ± 3.41	3.86 ± 2.41
**4**	8-F	NHBn	Bn	75.23 ± 0.63	2.92 ± 2.81
**5**	8-NO_2_	NHBn	Bn	86.38 ± 2.37	15.05 ± 0.55
**6**	7-OMe	NHBn	Bn	35.59 ± 2.28	33.47 ± 15.62
**7**	6-OMe	NHBn	Bn	43.86 ± 1.20	8.92 ± 2.87
**8**	8-OMe	indole-6-COOMe	Bn	82.10 ± 2.19	1.38 ± 0.73
**9**	8-OMe	pyrrolopyrmidine	Bn	91.59 ± 5.41	2.15 ± 0.09
**10**	8-OMe	naphthalen-1-yl	Bn	49.12 ± 2.89	3.97 ± 0.16
**11**	8-OMe	phenanthryl	Bn	64.95 ± 1.17	2.05 ± 0.32
**12**	8-OMe	2-methylnaphthyl	Bn	30.39 ± 0.43	10.42 ± 6.42
**13**	8-OMe	1-methylnaphthyl	Bn	28.27 ± 0.04	36.79 ± 0.89
**14**	8-OMe	NHBn 3-COOMe	Bn	42.10 ± 0.26	13.39 ± 9.03
**15**	8-OMe	NHBn 3-CF_3_	Bn	28.57 ± 1.13	25.61 ± 20.09
**16**	8-OMe	NHBn 3-OCF_3_	Bn	41.50 ± 2.98	7.52 ± 1.95
**17**	8-OMe	NHBn 3-CN	Bn	55.59 ± 2.13	82.61 ± 11.90
**18**	8-OMe	NHBn 4-Me	Bn	27.55 ± 0.01	48.09 ± 1.26
**19**	8-OMe	NHBn 4-OMe	Bn	29.98 ± 0.49	86.61 ± 22.39
**20**	8-OMe	NHBn 3-F	Bn	35.22 ± 0.66	99.48 ± 22.30
**21**	8-OMe	NHBn 4-OMe	Bn 4-OMe	33.98 ± 3.50	37.69 ± 13.03
**22**	8-OMe	NHBn 4-F	Bn 4-F	46.38 ± 2.33	51.23 ± 26.20

a Screened at 5 *μ*M. Data are mean ± SD from 2 independent experiments, each with technical triplicates.

b Screened at 1 *μ*M. Data are mean ± SD from at least 1 screen, each with technical triplicates.

**Table 2:** VCP ATPase inhibition of VPS4i screening hits


Compound	R^1^	R^2^	R^3^	VCP ATPase (% DMSO)^a^
4-107	8-OMe	NHBn	Bn	68.48 ± 2.85
**17**	8-OMe	NHBn 3-CN	Bn	91.83 ± 5.36
**18**	8-OMe	NHBn 4-Me	Bn	83.40 ± 4.64
**19**	8-OMe	NHBn 4-OMe	Bn	107.28 ± 2.66
**20**	8-OMe	NHBn 3-F	Bn	80.50 ± 0.57

a Screened at 5 *μ*M. Data are mean ± SD from 2 independent experiments, each with technical triplicates.

### 3.4 4-107 exhibits superior potency and selectivity for VPS4 in cells compared with DBeQ

**Fig. 4:**
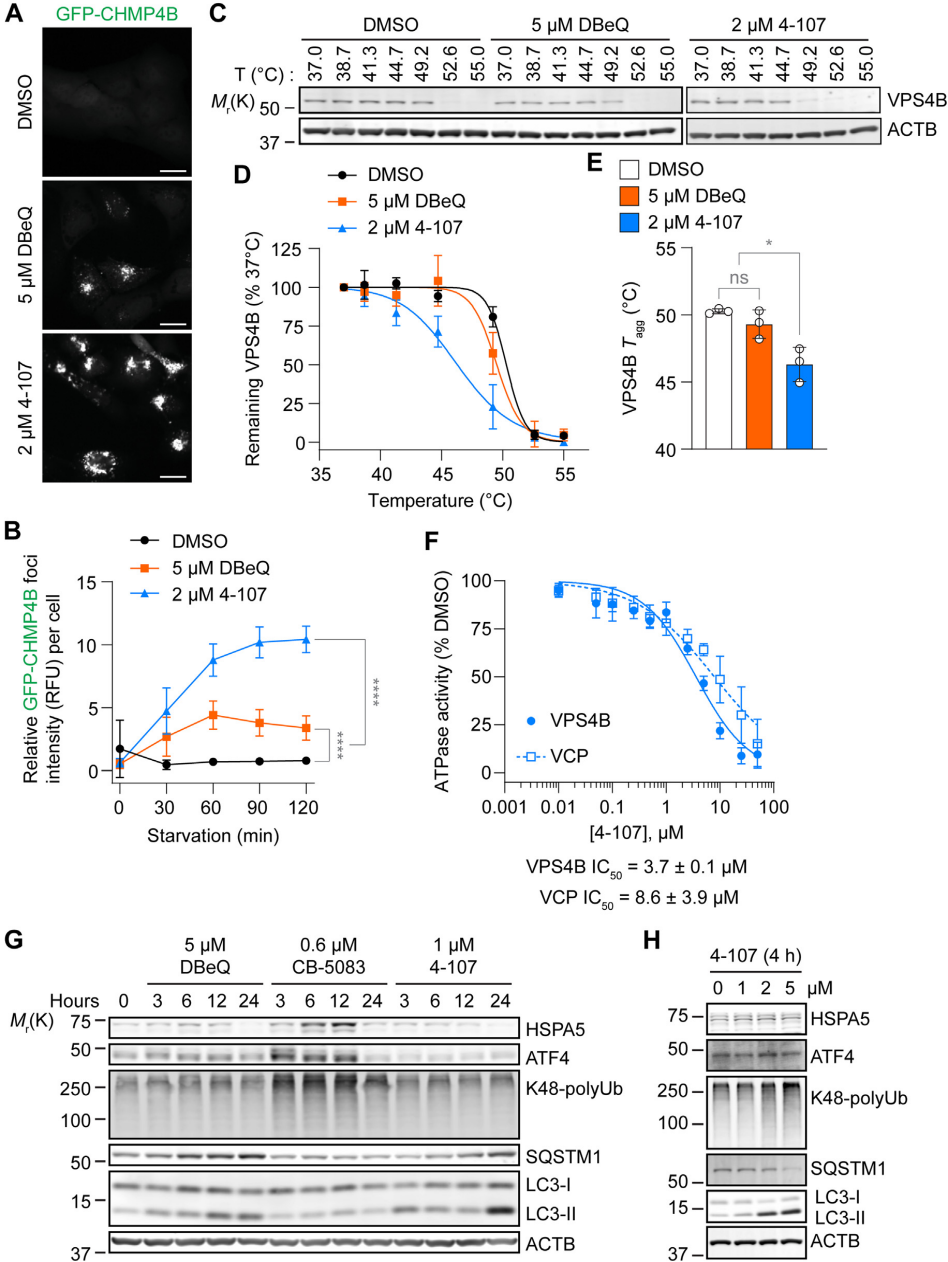
4-107 exhibits superior potency and selectivity for VPS4 in cells compared to DBeQ. (A) Representative HCM images of GFP-CHMP4B-expressing U2OS cells starved in the presence or absence of 5 *μ*M DBeQ and 2 *μ*M 4-107 for 2 hours. Scale bars: 20 *μ*m. (B) HCM quantification of relative fluorescence intensity of GFP-CHMP4B foci per cell from A. Data shown are representative of 2 independent experiments, each with twelve technical replicates. (C) Immunoblot analysis of soluble protein fractions from U2OS cells starved in the presence or absence of 5 *μ*M DBeQ and 2 *μ*M 4-107 for 1 hour followed by heating at the indicated temperatures. Representative blot shown from 3 independent experiments. (D) Quantification of relative VPS4B band intensities normalized to 37 °C from C. (E) VPS4B aggregation temperatures (*T*_agg_) from the temperature-response curves in D. (F) Remaining ATPase activities of VPS4B and VCP relative to DMSO when incubated with the indicated concentrations of 4-107. Data shown are from 3 independent experiments, each with technical triplicates. (G) Immunoblot analysis of U2OS cells treated in the presence or absence of 5 *μ*M DBeQ, 0.6 *μ*M CB-5083, and 1 *μ*M 4-107 for the indicated times in complete media. Representative blot shown from 3 independent experiments. (H) Immunoblot analysis of U2OS cells treated with the indicated concentrations of 4-107 for 4 hours in complete media. Representative blot shown from 3 independent experiments. All values in the graphs are mean ± SD, calculated using the mean values from each of the independent experiments. Statistical analysis was performed using one-way ANOVA followed by Tukey’s multiple comparison test; ns not significant, ∗*P* ≤ .05; ∗∗∗∗*P* < .0001.

Our findings showed 4-107 accumulated significantly more GFP-CHMP4B foci than DBeQ at approximately half the concentration, clearly demonstrating its increased potency (Fig. 4, A and B). These signals continually increased until reaching plateaus after about 1 hour of compound treatment. To confirm on-target activity, we evaluated intracellular engagement of VPS4B using the CETSA. This assay measures the change in protein aggregation temperature (*T*_agg_), analyzed via immunoblotting of the soluble protein fraction (Jafari et al, 2014). DBeQ induced a subtle thermal destabilization of VPS4B that was not statistically significant, whereas 4-107 decreased the VPS4B *T*_agg_ by approximately 3 °C, indicating that 4-107 binds more strongly to VPS4B intracellularly than DBeQ (Fig. 4C–E). These results correlate with the increased potency of 4-107 in GFP-CHMP4B accumulation, supporting that the 4-107 phenotype is related to improvement in-cell inhibition of VPS4.

Because DBeQ inhibits VCP cellular functions at the concentrations tested (Chou et al, 2011, 2013), and 4-107 was modestly selective for VPS4B in vitro (Fig. 4F, VPS4B IC_50_ = 3.7 ± 0.1 *μ*M, VCP IC_50_ = 8.6 ± 3.9 *μ*M), we assessed the cellular selectivity of 4-107 for VPS4- and VCP-mediated pathways. VPS4 is essential for autophagy and loss of VPS4 function inhibits autophagic flux (Takahashi et al, 2018; Zhen et al, 2019). Meanwhile, VCP is required for endoplasmic reticulum-associated degradation (ERAD; Rabinovich et al, 2002) where VCP inhibition results in the accumulation of ubiquitinated proteins and induction of ER stress (Weihl et al, 2006; Bastola et al, 2016). At a sublethal concentration (5 *μ*M), DBeQ mildly induced ER stress, based on the increase in HSPA5 and ATF4, and time-dependently inhibited autophagy, as evidenced by the elevated levels of SQSTM1 and LC3-II, supporting its activity against both ATPases (Fig. 4G). Potent VCP inhibitor CB-5083 (Zhou et al, 2015), used as a control for the VCP pathway, strongly induced ER stress, as well as the accumulation of K48-linked polyubiquitinated proteins, a marker of ERAD disruption and VCP inhibition (Locke et al, 2014; Zhou et al, 2015). Additionally, CB-5083 led to a reduction in SQSTM1, indicating the stimulation of autophagy as a consequence of ER stress (Ogata et al, 2006; Anderson et al, 2015). Notably, a sublethal dose of 4-107 (1 *μ*M) did not induce ER stress or the accumulation of polyubiquitinated proteins, unlike CB-5083. Instead, it inhibited autophagy over time, suggesting selective inhibition of VPS4-mediated pathways. This selective inhibition of the VPS4 pathway was also observed following a short-term (4 hours) treatment with 2 *μ*M 4-107 (Fig. 4H). However, at a higher dose (5 *μ*M), increases in ubiquitinated proteins and ATF4, along with a decrease in SQSTM1, were detected, indicating VCP pathway perturbation. Collectively, these results demonstrate the increased cellular potency and selectivity of 4-107 for VPS4 compared with DBeQ.

### 3.5 4-107 induces ESCRT accumulation on LC3-positive autophagosomal membranes

**Fig. 5:**
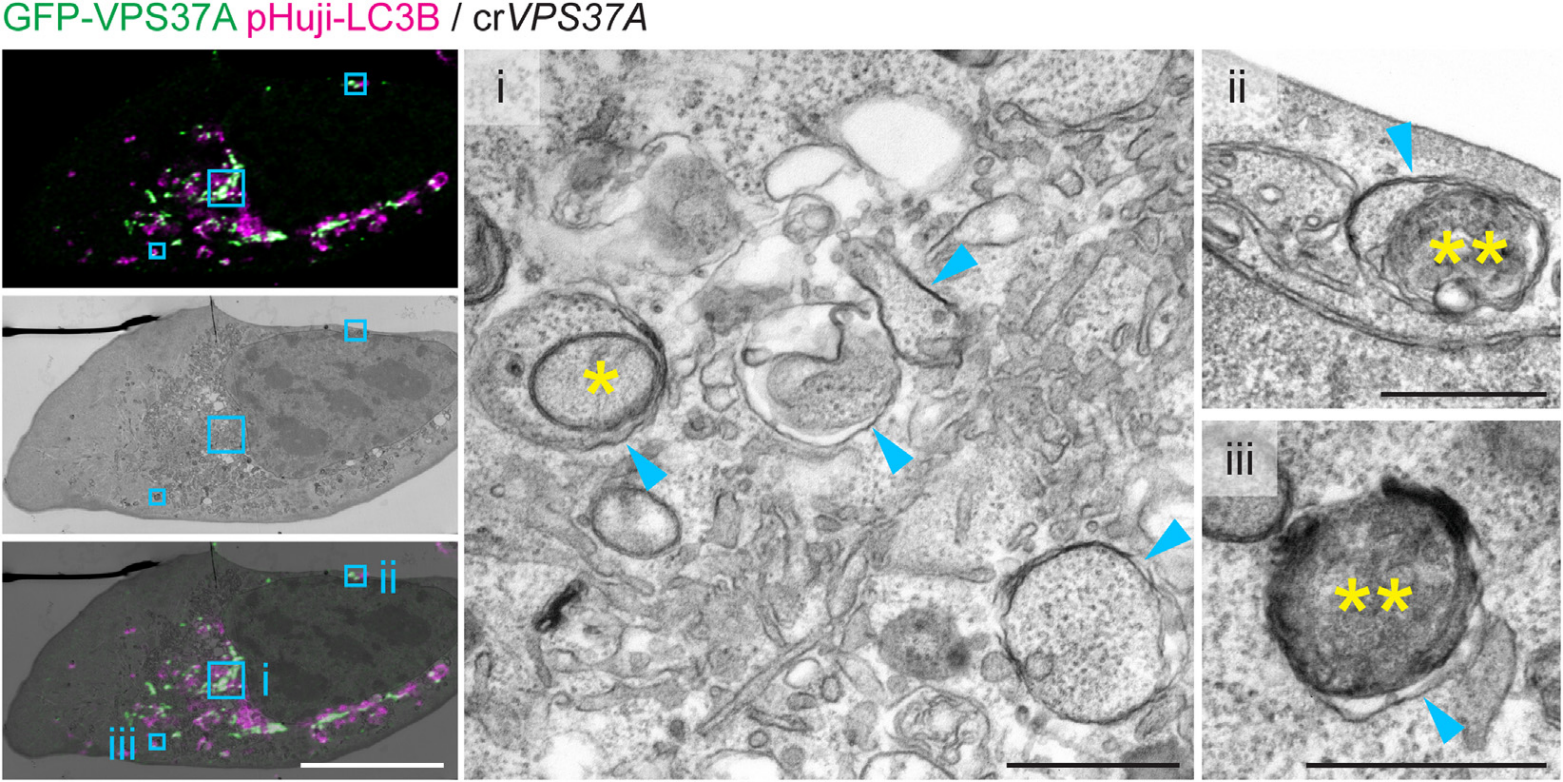
4-107 induces GFP-VPS37A accumulation on or near pHuij-LC3-positive autophagosomal membranes. Correlative light and electron microscopy (CLEM) analysis of GFP-VPS37A- and pHuij-LC3-expressing cr*VPS37A* U2OS cells starved in the presence of 1 *μ*M 4-107 for 2 hours. The left panels show confocal (top), electron microscopy (middle), and merged (bottom) images. Magnified images of VPS37A and LC3 double-positive structures in the box-indicated areas are shown in the middle and right panels. Asterisks, double-asterisks, and arrowheads indicate mitochondria, endolysosome-like, and autophagosome-like structures, respectively. Scale bars: 10 *μ*m (left) and 0.5 *μ*m (magnified images).

Because 4-107 accumulated GFP-CHMP4B in starved cells and inhibited autophagy, we sought to determine if 4-107 impairs ESCRT disassembly on autophagosomal membranes. We have previously found that VPS4 inhibition results in the membrane accumulation of not only ESCRT-III but also upstream ESCRT components, including the ESCRT-I subunit VPS37A, during autophagy (Takahashi et al, 2019). Consistently, the accumulation of GFP-VPS37A signals on pHuji-LC3B-labeled autophagosomal membranes was detected during starvation in the presence of 4-107 (Fig. 5). Ultrastructural analysis confirmed that these structures were immature autophagosomal membranes containing cytoplasmic materials, including mitochondria. Interestingly, the engulfment of endolysosomal structures was also observed by GFP-VPS37A and pHuji-LC3B double-positive structures, resembling simaphagy induced upon the inhibition of ESCRT-mediated ubiquitinated receptor turnover (Migliano et al, 2023) or lysophagy for damaged endolysosome clearance (Maejima et al, 2013; Kakuda et al, 2023).

### 3.6 4-107 exhibits lysosomotropic properties and disrupts endolysosomal membrane integrity

**Fig. 6:**
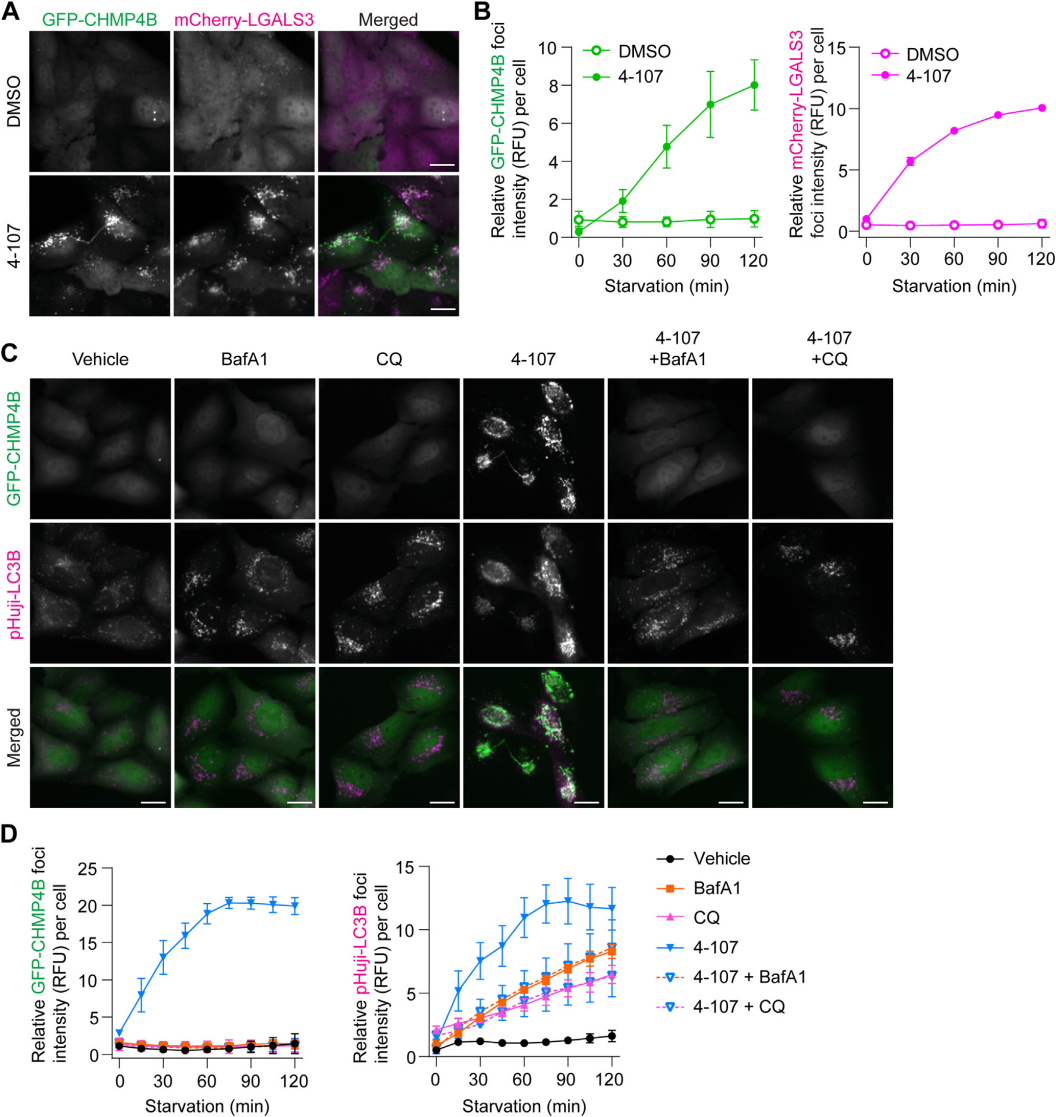
4-107 exhibits lysosomotropic properties and disrupts endolysosomal membrane integrity. (A) Representative HCM images of GFP-CHMP4B- and mCherry-LGALS3-expressing U2OS cells starved in the presence or absence of 2 *μ*M 4-107 for 2 hours. (B) HCM quantification of relative fluorescence intensities of GFP-CHMP4B (left) and mCherry-LGALS3 (right) foci per cell from A. Data shown are representative of 4 independent experiments, each with technical triplicates. (C) Representative HCM images of GFP-CHMP4B- and pHuji-LC3B-expressing U2OS cells starved in the presence or absence of 100 nM BafA1, 50 *μ*M CQ, 2 *μ*M 4-107, or 4-107 with each BafA1 and CQ for 2 hours. (D) HCM quantification of relative fluorescence intensities of GFP-CHMP4B (left) and pHuji-LC3B (right) foci per cell from C. Data shown are representative from 3 independent experiments, each with technical triplicates. All values in the graphs are mean ± SD, calculated using the mean values from each of the independent experiments. Scale bars: 20 *μ*m.

Because membrane sequestration of endolysosomes was observed in cells treated with 4-107 (Fig. 5), we investigated if 4-107 affects endolysosomal membrane integrity by monitoring the accumulation of galectin 3 (LGALS3), which recognizes lumenal glycoproteins exposed upon membrane damage (Paz et al, 2010; Maejima et al, 2013). We found that the time-dependent accumulation of GFP-CHMP4B was accompanied by an increase in mCherry-LGALS3 signals, indicating disruption of membrane integrity (Fig. 6, A and B). Notably, both CHMP4B and LGALS3 signals did not decrease during the time course examined, suggesting defects in ESCRT-mediated membrane repair in the presence of 4-107. Given that 4-107 disrupted endolysosomal membrane integrity, we next investigated whether the compound exhibited lysosomotropic properties by co-treating with lysosomal inhibitors BafA1 or CQ, both of which neutralize the acidic endolysosomal pH. We observed that 4-107-induced GFP-CHMP4B accumulation was blocked by both BafA1 and CQ, whereas the 4-107-induced increase in pHuji-LC3B foci was lowered to that seen with the pH-neutralizing agents alone (Fig. 6, C and D). Altogether, these results suggest 4-107 exhibited lysosomotropic properties, induced endolysosomal damage, and disrupted ESCRT turnover for membrane repair.

### 3.7 4-107 induces CASP8-mediated VPS4-sensitive cell death

**Fig. 7:**
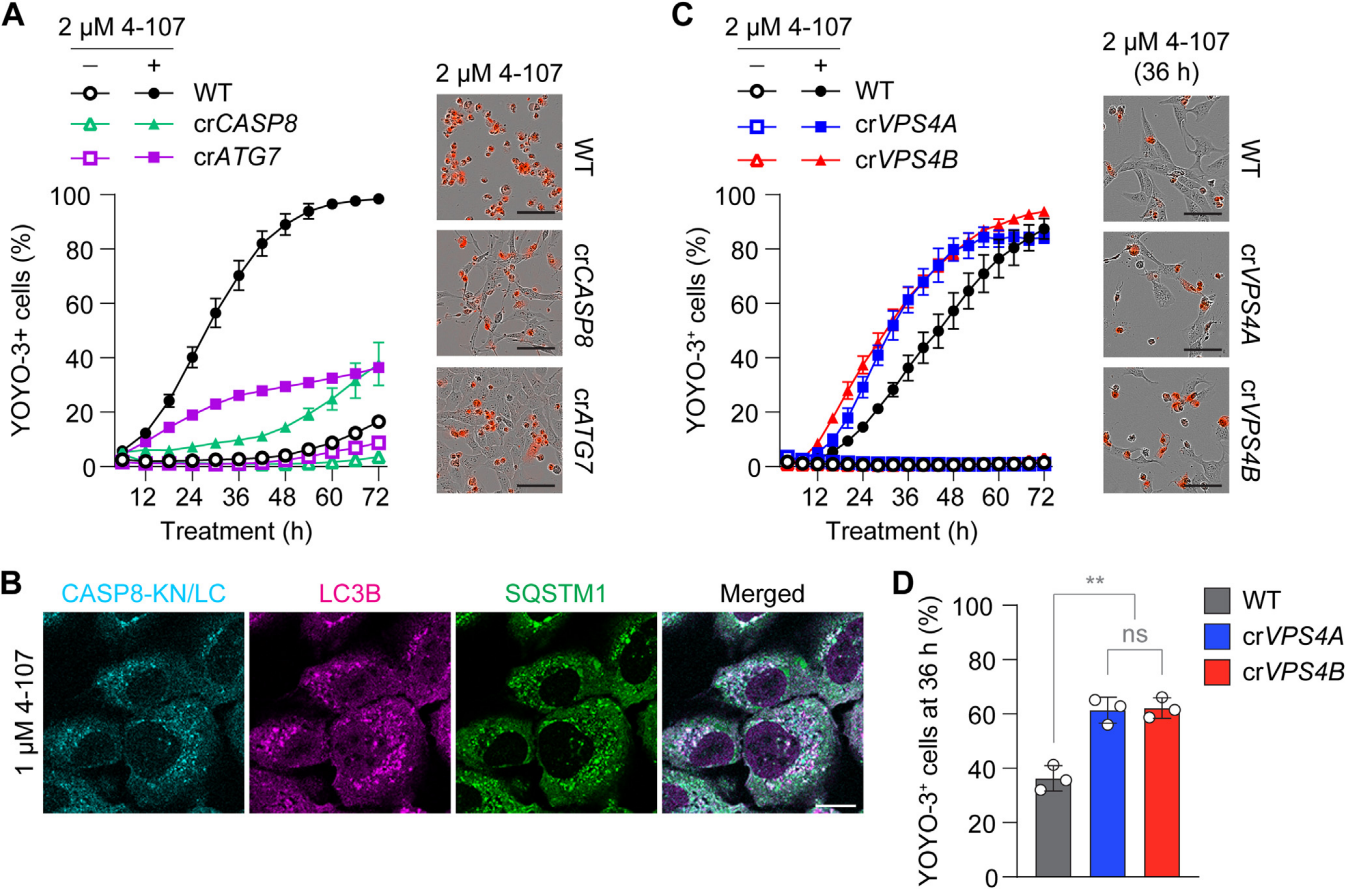
4-107 induces CASP8-mediated VPS4-sensitive cell death. (A) Live-cell quantification of YOYO-3-positive dead WT, cr*CASP8*, and cr*ATG7* U2OS cells in the presence or absence of 2 *μ*M 4-107. Representative images of cells at experimental endpoint are shown Data shown are representative of 3 independent experiments, each with technical triplicates. (B) Confocal images of CASP8^C360A^-KN151/LC151-expressing U2OS cells starved in the presence of 1 *μ*M 4-107 for 2 hours, then stained for LC3B and SQSTM1. (C) Live-cell quantification of YOYO-3-positive dead WT, cr*VPS4A*, and cr*VPS4B* U2OS cells in the presence or absence of 2 *μ*M 4-107. Representative images of cells at 36 hours are shown. Data shown are representative of 3 independent experiments, each with technical triplicates. (D) Live-cell quantification of YOYO-3-positive dead cells from C at 36 hours. Data shown are mean ± SD calculated using the mean values from each of the 3 independent experiments shown in C. Scale bars: 100 *μ*m in A and C, 10 *μ*m in B. Statistical analysis was performed using one-way ANOVA followed by Tukey’s multiple comparison test; ns not significant; ∗∗*P* < .01.

As 4-107 disrupted ESCRT function, we examined whether the compound could induce CASP8-dependent cell death similar to the genetic inhibition of VPS4 (Figs. 1 and 2). We found that 4-107 treatment induced cell death in a time-dependent manner, which was inhibited by the loss of not only *CASP8* but also *ATG7* (Fig. 7A), suggesting that 4-107 can trigger iDISC-mediated cell death. To verify this, we observed the accumulation of pro-CASP8 dimers by the bimolecular fluorescence complementation (BiFC) assay as previously described (Young et al, 2012). As expected, pro-CASP8 BiFC signals accumulated on LC3- and SQSTM1-positive autophagic structures upon 4-107 treatment (Fig. 7B). We then explored if 4-107 could provoke synthetic lethality in VPS4 paralog-deficient cells. Compared with WT cells, *VPS4A*- and *VPS4B*-deficient cells displayed enhanced temporal sensitivity toward 4-107 by about 12 hours (Fig. 7, C and D). Collectively, these findings indicate that 4-107 induces cell death in a VPS4-sensitive manner by triggering iDISC-mediated apoptosis and exhibiting greater toxicity to cells with reduced VPS4 levels.

### 3.8 4-107 suppresses tumor growth in vivo

**Fig. 8:**
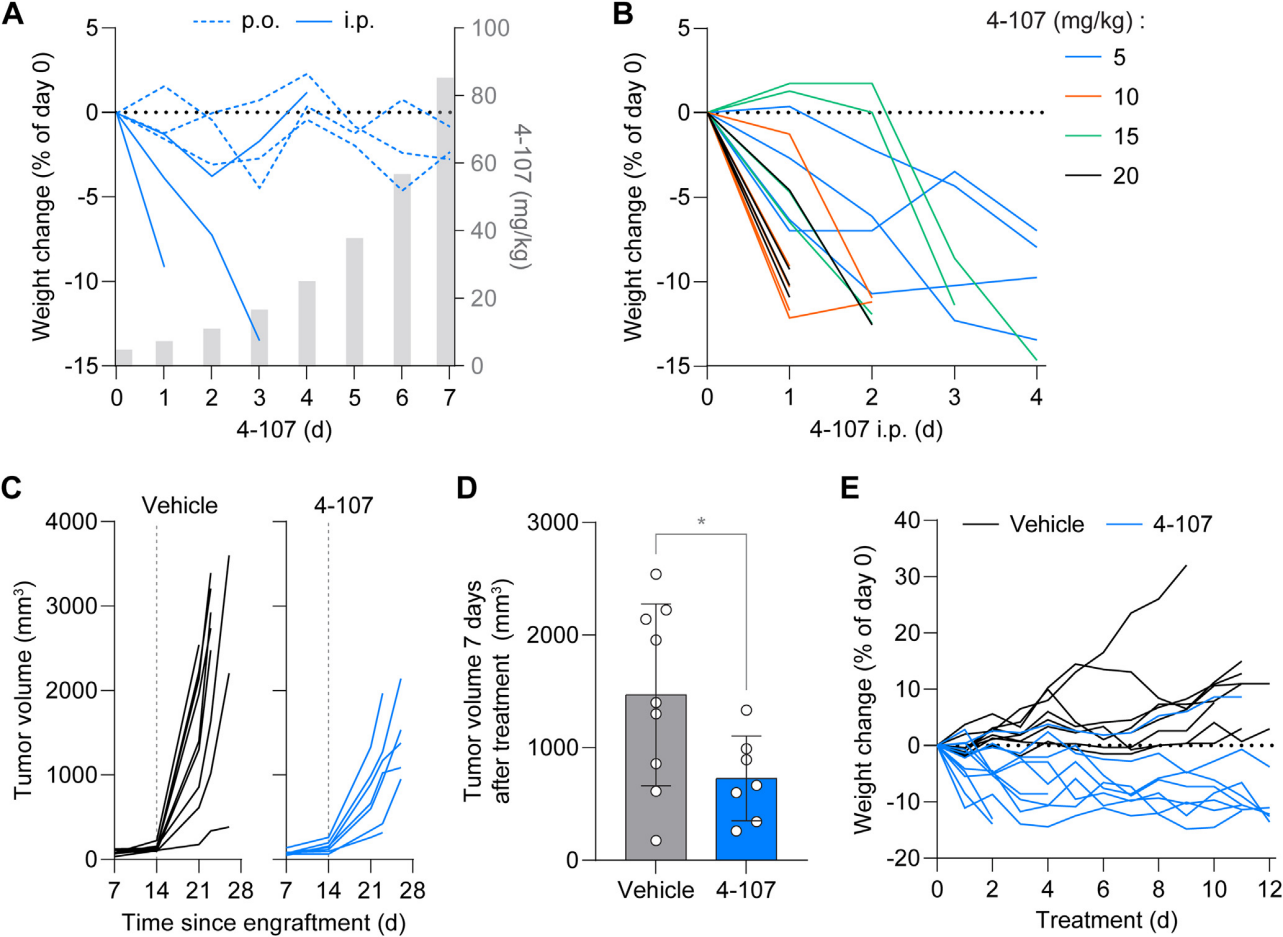
4-107 suppresses tumor growth in vivo. (A) Body weight changes relative to day 0 (left axis) of mice administered 4-107 orally (by mouth; 3 mice) or intraperitoneally (3 mice) daily with doses increasing 50% per day (right axis). (B) Body weight changes relative to day 0 of mice administered 4-107 i.p. daily at the indicated doses (5 and 10 mg/kg, 4 mice each; 15 and 20 mg/kg, 5 mice each). (C) Volume of 9464D s.c. tumors in B6 mice administered vehicle (9 mice) or 5 mg/kg 4-107 i.p. daily (10 mice). The first day of treatment (day 14) is indicated by dashed lines. (D) Tumor volumes after 7 days of treatment from C. (E) Body weight changes relative to day 0 for mice from C. Statistical analysis was performed using unpaired two-tailed *t* test; ∗*P* ≤ .05.

To assess the antitumor effects of 4-107 in vivo, we first subjected 4-107 to a panel of standard in vitro ADME assays (Table 3). 4-107 exhibited notable human liver microsomal stability and substantial human plasma protein binding. On the other hand, it displayed poor intestinal fluid solubility and minimal Caco-2 monolayer permeability with probable efflux, suggesting that the compound would have poor oral bioavailability. Next, we performed MTD studies of 4-107 in mice to determine the optimal dosing and the route of administration. We began with an escalating dose MTD study, administering 4-107 either orally or intraperitoneally, with daily doses increasing by 50% each day. Although 4-107 was well tolerated via the oral route at doses up to approximately 90 mg/kg, the intraperitoneal route exhibited toxicity at doses above 25 mg/kg (Fig. 8A). The lack of weight loss with oral administration even at the highest doses, contrasted with the intolerance of intraperitoneal administration at 4-fold lower doses, suggests limited absorption and is consistent with its predicted poor oral bioavailability (Table 3). As a result, we selected the intraperitoneal route for further studies and determined the acute dose MTD of 4-107 by administering fixed daily doses with a maximum of 20 mg/kg and found that 5 mg/kg was tolerated by the mice (Fig. 8B). Finally, we examined the efficacy of 4-107 on tumor growth. As U2OS cells exhibited limited tumor formation in mice, we used the 9464D syngeneic mouse model of neuroblastoma, where tumor growth depends on the functional ESCRT and autophagy machinery (Zhang et al, unpublished data). Treatment with 4-107 (5 mg/kg/day, i.p.) significantly reduced tumor growth (Fig. 8, C and D) without causing severe toxicity in most mice (7 of 10 mice tolerated; Fig. 8E). These results demonstrate that 4-107 exhibited antitumor activity in a neuroblastoma mouse model.

**Table 3:** In vitro ADME properties of 4-107

Assay	Result
Thermodynamic solubility^a^	17.0 *μ*M
Caco-2 permeability A-B, P_app_^b^	0.03 × 10^−6^ cm/s
Caco-2 permeability B-A, P_app_^b^	0.30 × 10^−6^ cm/s
Caco-2 permeability efflux ratio	10.29
Liver microsomal stability, remaining after 1 h^c^	71.83%
Liver microsomal stability, T_1/2_^c^	117.1 min
In vitro intrinsic clearance, Cl_int_^c^	11.8 *μ*L/min/mg
Plasma protein binding^d^	99.93%

a In FaSSIF-V2, pH 6.5.

b 10 *μ*M of compound.

c Human liver microsomes, 1 *μ*M of compound.

d Human plasma, 5 *μ*M of compound. Mean values are displayed with respective units.

## 4 Discussion

Targeting VPS4 is an attractive approach for cancer therapy as various types of cancers are dependent on either *VPS4A* or *VPS4B* for survival (McDonald et al, 2017; Neggers et al, 2020; Szymańska et al, 2020). In this study, we investigated the mechanisms underlying VPS4 inhibition-induced cell death and developed 4-107, a small molecule that inhibited VPS4 ATPase activity in vitro and exhibited antitumor activity in vivo. The observations that the genetic inhibition of VPS4, either by DN VPS4A overexpression or concurrent loss of *VPS4A* and *VPS4B*, activated the CASP8-initiated caspase cascade align with that of our previous studies demonstrating that *CHMP2A* depletion triggers noncanonical CASP8 activation on autophagosomal membranes, leading to apoptosis induction (Hattori et al, 2021, 2023). However, unlike *CHMP2A* depletion, we found that neither the loss of *ATG5*, which impairs CASP8 recruitment to autophagosomal membranes (Young et al, 2012), nor caspase inhibition was sufficient to prevent cell death induced by VPS4 genetic inhibition. A recent study suggests the implications of multiple cell death pathways for *VPS4* loss-induced cell death, including caspase-independent, RIPK1-mediated cell death in addition to apoptosis (Szymańska et al, 2020). Unlike autophagosome closure, which selectively requires CHMP2A (Takahashi et al, 2018), in several other ESCRT-mediated membrane remodeling processing, including HIV budding (Morita et al, 2011) and endolysosomal membrane repair (Chen et al, 2019), the loss of CHMP2A can be, at least in part, compensated by its paralog, CHMP2B. Moreover, VPS4 also exhibits several ESCRT-III-independent functions, such as cholesterol trafficking and centrosome positioning (Du et al, 2013; Ott et al, 2018). Thus, the activation of CASP8-independent cell death pathways observed upon VPS4 genetic inhibition could be attributed to the broader functions of VPS4 compared with CHMP2A.

We found that the lead compound 4-107, which was previously reported during the SAR optimization of DBeQ for VCP inhibition (Chou et al, 2013), exhibited inhibitory activity against VPS4 in addition to VCP in vitro. However, in cells, 4-107 primarily disturbed VPS4-dependent processes, including autophagy and the maintenance of lysosomal membrane integrity, but not the VCP-dependent ERAD pathway at the doses tested. The precise mechanism behind the enhanced in-cell potency of 4-107 toward these VPS4-mediated pathways remains unclear but could be attributed to its lysosomotropic properties. The observation that 4-107 caused lysosomal damage supports the notion that quinazolinamine class compounds, such as prazosin, tend to accumulate in endolysosomes leading to membrane permeation (Kozik et al, 2020). Notably, although the ESCRT machinery is crucial for the repair and removal of damaged endolysosomes (Maejima et al, 2013; Radulovic et al, 2018; Skowyra et al, 2018), we found that CHMP4B, LGALS3, and LC3 signals were continuously accumulated during the course of 4-107 exposure, indicating the impairment of ESCRT disassembly, membrane repair, and autophagic clearance by the treatment. However, given that these phenotypes were reversed in the presence of lysosomal inhibitors, it is unlikely that superior potency and selectivity toward VPS4 over VCP is the main contributor to the observed in-cell selectivity of 4-107. Rather, we suggest that disruption of lysosomal membrane integrity increases ESCRT recruitment to damaged endolysosomes, enabling the local enhancement of VPS4 inhibition and the subsequent disruption of ESCRT-mediated membrane repair and clearance.

Our results showed that 4-107 triggers CASP8-mediated apoptosis, which is further enhanced by the loss of either *VPS4A* or *VPS4B*. This pro-death effect of 4-107 is not simply due to the accumulation of damaged endolysosomes, which could activate CASP8 via releasing lysosomal cathepsins (Zou et al, 2013), given that the disruption of LC3 conjugation and autophagy by *ATG7* loss did not enhance but rather abrogated 4-107-induced cell death. Furthermore, an accumulation of pro-CASP8 dimers on LC3 and SQSTM1 double-positive structures was detected in cells treated with 4-107. These findings support the notion that 4-107 treatment induces local VPS4 inhibition at sites of damaged endolysosomes, stabilizing nearby autophagosomal membranes to facilitate the assembly and activation of iDISCs, thereby inducing apoptosis. Importantly, the cells deficient in either *VPS4A* or *VPS4B* were found to be more susceptible to 4-107-induced cell death. Given that cells depleted of one *VPS4* paralog have been shown to be more vulnerable to endolysosomal rupture (Chen et al, 2019), 4-107-like compounds exhibiting VPS4 inhibitory activity with lysosomotropic properties may be effective against cancer cells with *VPS4* copy number loss by enhancing CASP8-mediated apoptosis.

The observation that 4-107 inhibited tumor growth without causing severe toxicity aligns with previous findings that cancer cells are more susceptible to ESCRT inhibition compared with noncancerous cells (Hattori et al, 2021). To the best of our knowledge, this study provides the first preclinical evidence demonstrating the potential of VPS4 inhibitors for cancer therapy. However, at higher doses or with prolonged treatment, significant weight loss was observed in treated mice. Because 4-107 also exhibited inhibitory activity against VCP in vitro and lysosomotropic properties in cells, these off-target effects may contribute to the observed toxicity. Additionally, it is conceivable that similar to cancer cells with *VPS4A* or *VPS4B* LOH, cells in certain tissues may depend on 1 *VPS4* paralog due to the limited expression of the other. Indeed, although VPS4A and VPS4B have been considered to function redundantly in mediating various ESCRT-dependent membrane remodeling processes (Scheuring et al, 2001; Beyer et al, 2003; Neggers et al, 2020), mice systemically disrupted for either paralog exhibit embryonic or postnatal lethality (Drusenheimer et al, 2015; Cacheiro et al, 2020; Chen et al, 2021). It would be interesting to determine if 4-107 induced cell death, enhanced by the loss of either *VPS4* paralog, allows for lowering effective inhibitory doses and mitigating its side effects. Nonetheless, further optimization of 4-107 is warranted to improve its potency, selectivity, and pharmacokinetic properties.

## Conflict of interest

The authors declare no conflicts of interest.
