# Supplementary material for: DBeQ derivative targets vacuolar protein sorting 4 functions in cancer cells and suppresses tumor growth in mice
Source: J Pharmacol Exp Ther. 2025 Feb 28;392(4):103524. doi: 10.1016/j.jpet.2025.103524 (PMC13095408; doi:10.1016/j.jpet.2025.103524)
Supplement: Supplementary Tables 1-3 [file mmc1.docx]

**JPET-D-24-00055**

**Journal of Pharmacology and Experimental Therapeutics**

**Supplemental Material**

**DBeQ derivative targets VPS4 functions in cancer cells and suppresses tumor growth in mice**

Kevin A. Fundora, Yan Zhuang, Kouta Hamamoto, Guifang Wang, Longgui Chen, Tatsuya Hattori, Xinwen Liang, Lei Bao, Venugopal Vangala, Fang Tian, Yoshinori Takahashi*, and Hong-Gang Wang*

Division of Pediatric Hematology and Oncology, Department of Pediatrics, The Pennsylvania State University College of Medicine, Hershey, Pennsylvania (K.A.F., Y.Z., K.H., L.C., T.H., X.L., L.B., V.V., Y.T., H.-G.W.)

Department of Biochemistry and Molecular Biology, The Pennsylvania State University College of Medicine, Hershey, Pennsylvania (G.W., F.T.)

Department of Pharmacology, The Pennsylvania State University College of Medicine, Hershey, Pennsylvania (T.H., H.-G.W.)

*Correspondence to: Yoshinori Takahashi, [ytakahashi@pennstatehealth.psu.edu](mailto:ytakahashi@pennstatehealth.psu.edu); Hong-Gang Wang, [huw11@psu.edu](mailto:huw11@psu.edu); Department of Pediatrics, The Pennsylvania State University College of Medicine, 500 University Drive, Hershey, PA 17033

**Supplemental Table 1. Oligonucleotides used for plasmid construction.**

| Name | Sequence (5'-3') | Purpose | Reference |
| --- | --- | --- | --- |
| sg*ATG5* | AAGATGTGCTTCGAGATGTG | lentiCRISPR v2-sgATG5 | This study |
| sg*VPS4A* | CAACATACGGTGGAACGACG | epiCRISPR-sgVPS4A |  |
| sg*VPS4B* | CCACTTAGAAACAAGATCAG | epiCRISPR-sgVPS4B |  |
| VPS4B F | GGATCCATGTCATCCACTTCGCCCAAC | pET28a-VPS4B |  |
| VPS4B R | CTCGAGTTAGCCTTCTTGACCAAAATCTTC |  |  |
| pHuji F | TATATCGCTAGCATGGTGAGCAAGGGCG | pCDH-CMV-pHuji-LC3B-SV40-Hygro |  |
| pHuji R | TCTAGACTCGAGCTTGTACAGCTCGTCCAT |  |  |
| mEGFP-CHMP4B F | ATGGTGAGCAAGGGCGAGGAG | pCDH-CMV-pHuji-LC3B-tPT2A-GFP-CHMP4B-SV40-Hygro |  |
| mEGFP-CHMP4B R | GTACCACCACACTGGGATCCTTACATGGATCCAGCCCAG |  |  |
| mCherry-Galectin3 F | TCGACTCTAGAGGATCCGCTAGCGCTACCGGTCGCCACCATGGTGAGCAAGGGCGAGG | pCDH-UbC-mCherry-LGALS3-SV40-Bleo |  |
| mCherry-Galectin3 R | TACCGTCGACTGCAGAATTCTTATATCATGGTATATGAAGCACTGGTG |  |  |
| sh*Seed* | CGAGAAGCTGTTCGATTATTT | pRSITEP-U6Tet-shRNA-EF1-TetRep-2A-Puro | PMID: 33326793 |
| sh*VPS4A* | CGAGAAGCTGAAGGATTATTT |  |  |

**Supplemental Table 2. Compounds in this study.**

| Cmpd | Structure | VPS4B ATPase  (% DMSO) ^a^ | GFP-CHMP4B  (% 4-107) ^b^ | VCP ATPase  (% DMSO) ^c^ |
| --- | --- | --- | --- | --- |
| DBeQ * | 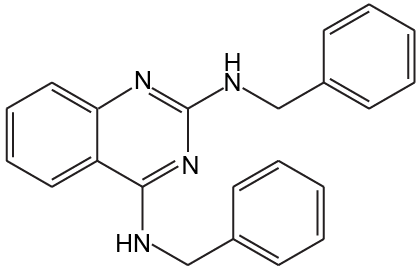 | 41.96 ± 7.53 | 8.93 ± 3.51 | 74.64 ± 6.01 |
| 4-95 | 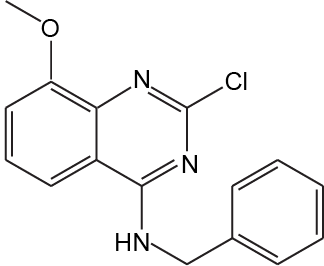 |  | 1.76 ± 0.74 |  |
| 4-107 (#1) | 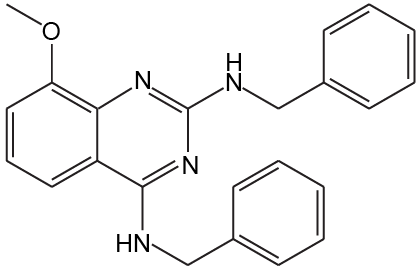 | 36.67 ± 1.73 | 100.00 ± 17.20 | 68.48 ± 2.02 |
| 4-127 (#17) | 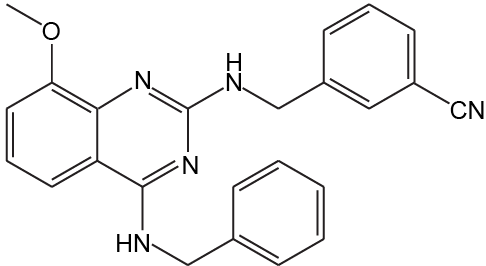 | 55.59 ± 2.13 | 82.61 ± 11.90 | 91.83 ± 3.79 |
| 4-131-iii | 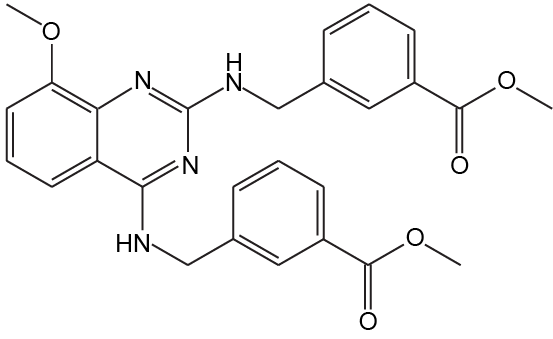 | 34.70 ± 0.70 | 11.94 ± 9.74 |  |
| 4-131-iv | 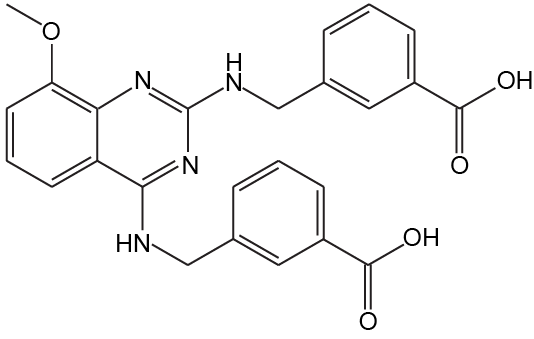 | 105.12 ± 0.39 | 1.10 ± 0.68 |  |
| 5-03 | 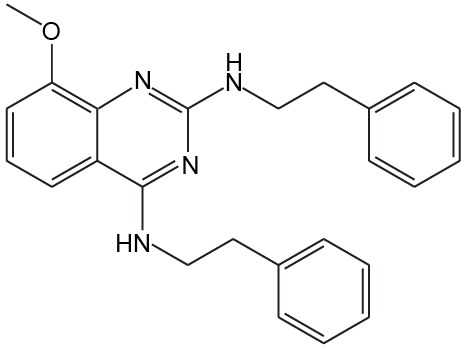 | 30.96 ± 1.37 | 2.31 ± 1.75 |  |
| 5-05 (#22) | 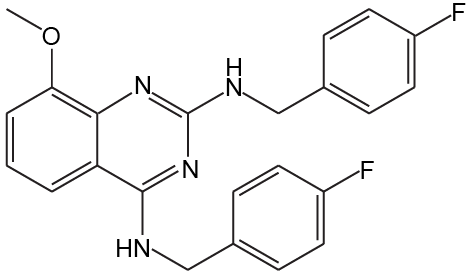 | 46.38 ± 2.33 | 51.23 ± 26.20 | 99.33 ± 2.70 |
| 5-07 | 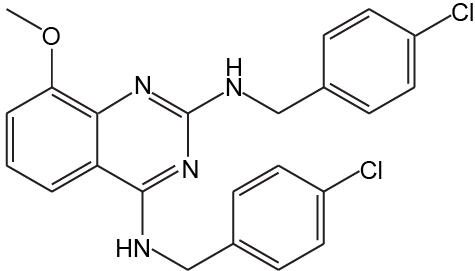 | 28.5 ± 0.04 | 14.56 ± 10.71 |  |
| 5-09 (#21) | 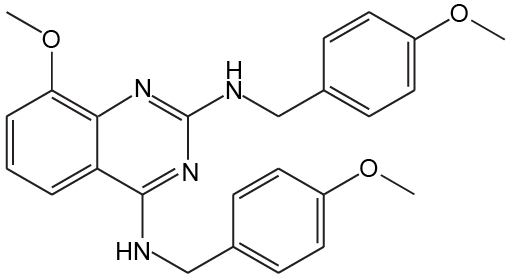 | 33.98 ± 3.50 | 37.69 ± 13.03 | 100.96 ± 0.28 |
| 5-11 | 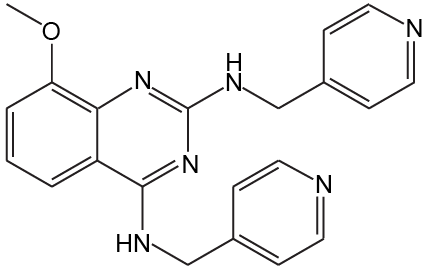 | 82.46 ± 1.64 | 1.10 ± 0.76 |  |
| 5-15-iv | 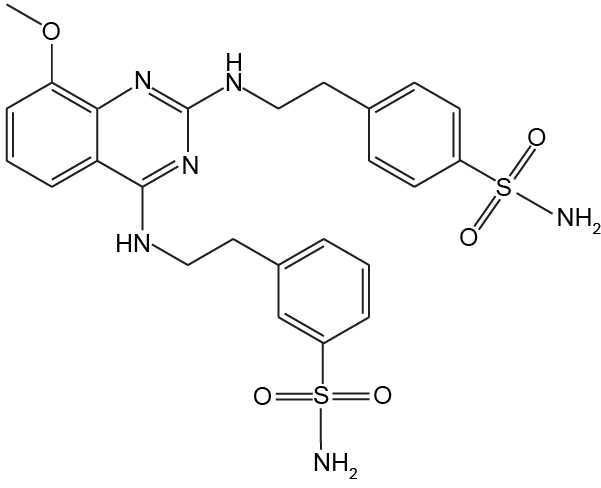 | 77.37 ± 4.37 | 3.22 ± 0.67 |  |
| 5-17 | 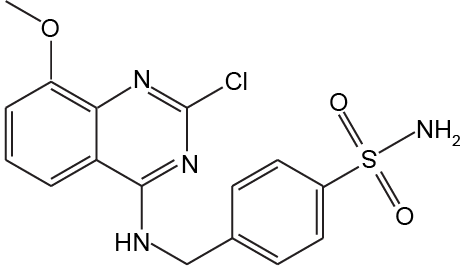 | 86.08 ± 7.25 | 2.87 ± 0.27 |  |
| 5-21 (#19) | 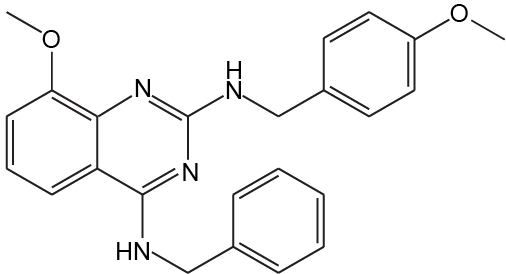 | 29.98 ± 0.49 | 86.61 ± 22.39 | 107.28 ± 1.88 |
| 5-27-2 | 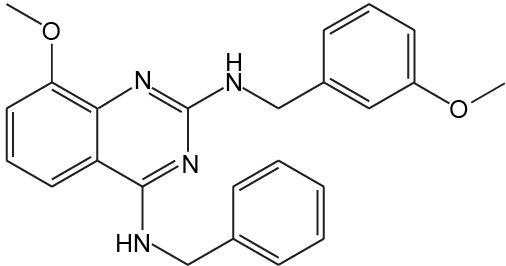 | 51.50 ± 0.83 | 71.11 ± 0.37 | 100.03 ± 0.81 |
| 5-33-iv | 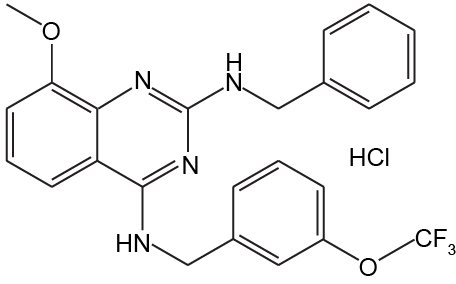 | 25.77 ± 2.48 | 5.27 ± 1.53 |  |
| 5-35-iii (#14) | 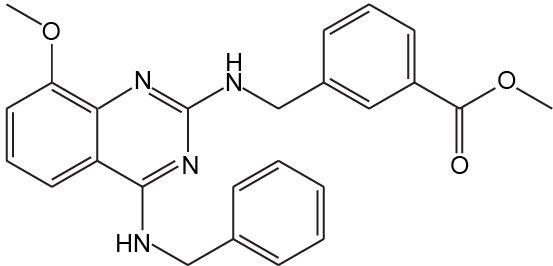 | 42.10 ± 0.26 | 13.39 ± 9.03 |  |
| 5-35-iv | 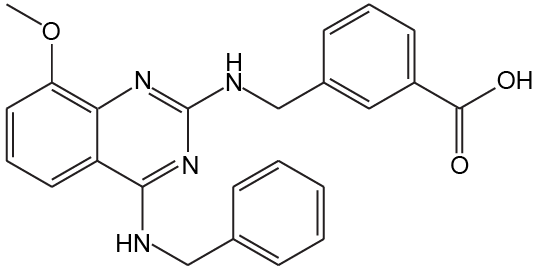 | 88.20 ± 5.24 | 10.92 ± 9.21 |  |
| 5-39-iii (#8) | 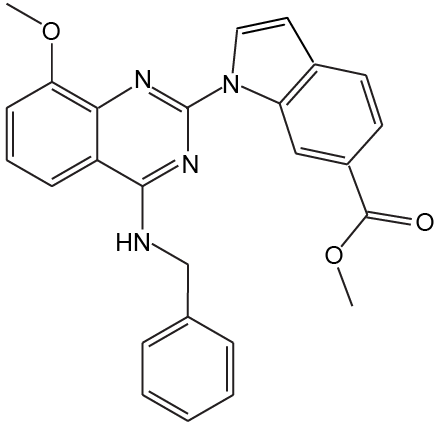 | 82.10 ± 2.19 | 1.38 ± 0.73 |  |
| 5-39-iv | 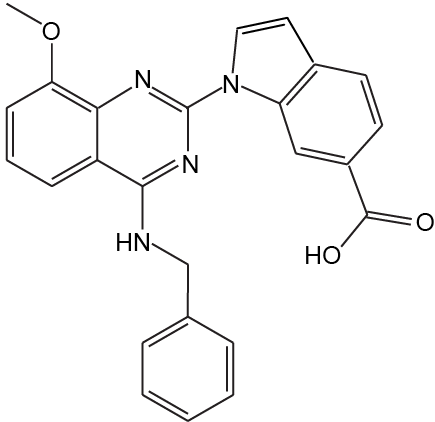 | 93.04 ± 0.65 | 1.59 ± 0.93 |  |
| 5-41-iii | 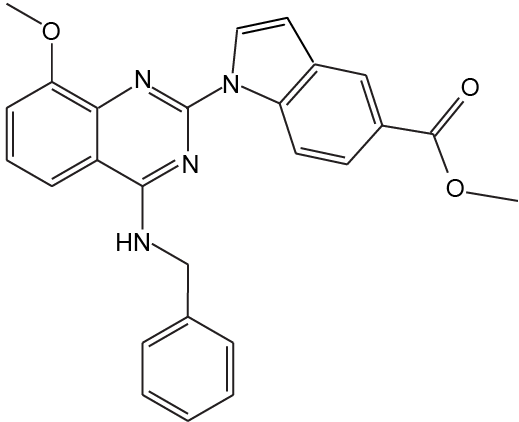 | 80.70 ± 2.02 | 2.82 ± 1.33 |  |
| 5-41-iv | 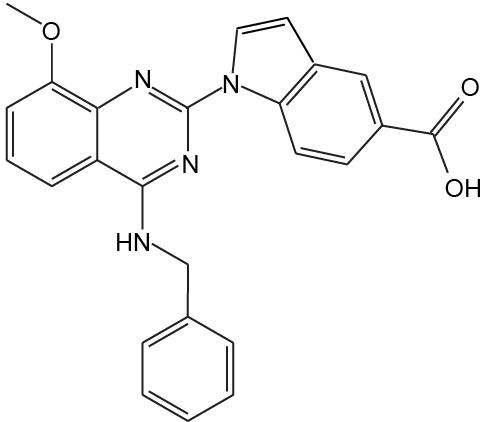 | 87.4 ± 1.43 | 1.29 ± 1.06 |  |
| 5-43-iii | 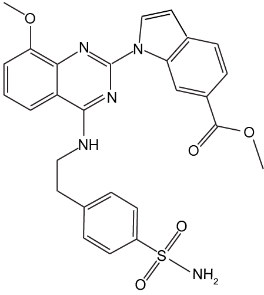 | 94.98 ± 0.53 | 3.01 ± 1.98 |  |
| 5-43-iv | 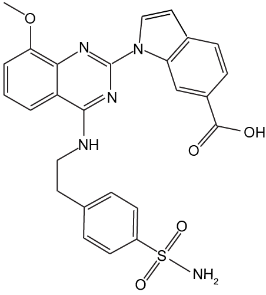 | 99.19 ± 4.43 | 1.27 ± 0.69 |  |
| 5-47 | 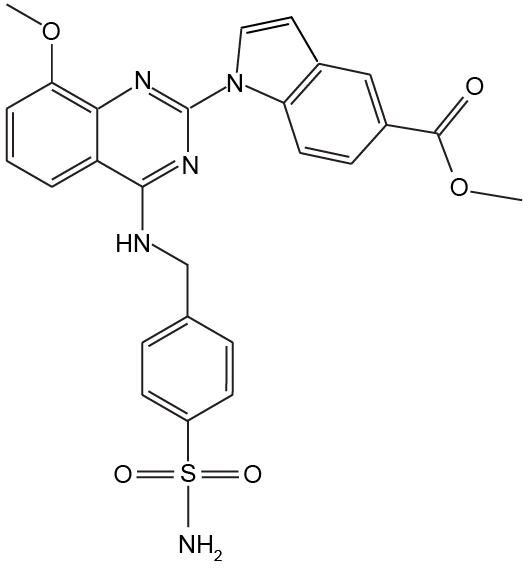 |  | 1.41 ± 0.61 |  |
| 5-49 (#9) | 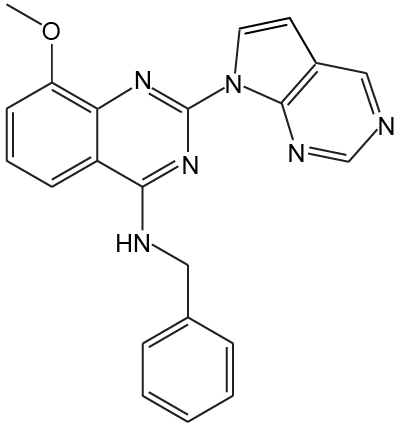 | 91.59 ± 5.41 | 2.15 ± 0.09 |  |
| 5-51 | 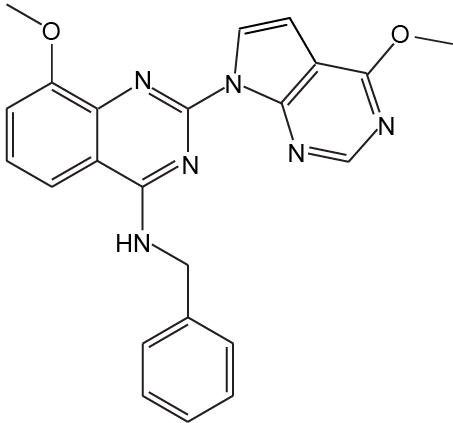 | 91.51 ± 3.26 | 2.51 ± 1.24 |  |
| 5-53 | 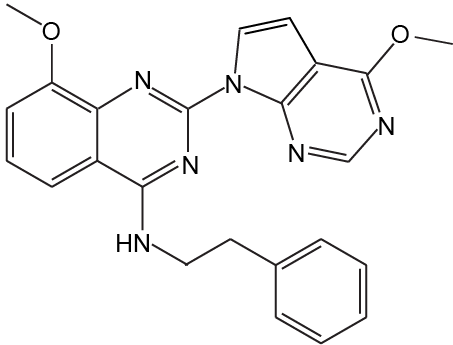 | 72.71 ± 0.81 | 1.68 ± 0.33 |  |
| 5-55 | 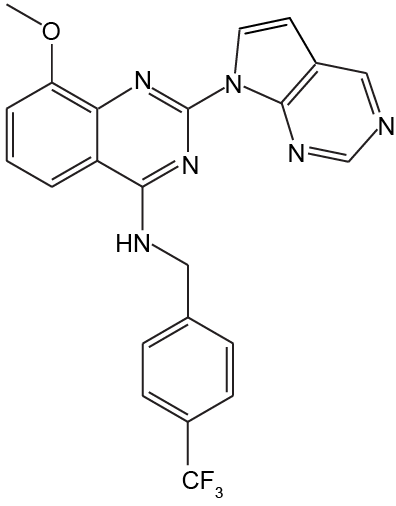 | 93.20 ± 1.22 | 2.25 ± 0.55 |  |
| 5-57 | 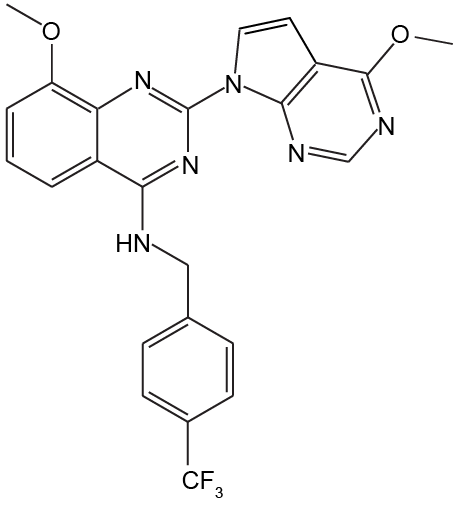 | 93.98 ± 3.83 | 2.44 ± 0.81 |  |
| 5-59 | 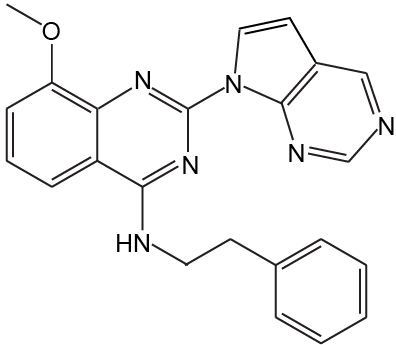 | 97.54 ± 1.15 | 2.84 ± 0.06 |  |
| 5-63 | 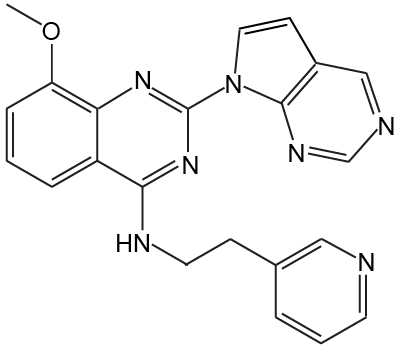 | 90.35 ± 0.07 | 5.28 ± 0.27 |  |
| 5-65 | 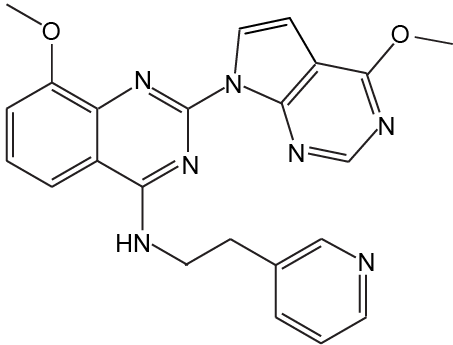 | 89.36 ± 2.84 | 1.69 ± 0.46 |  |
| 5-67 | 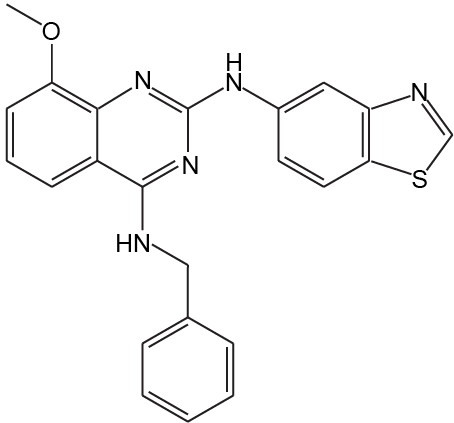 | 51.60 ± 0.35 | 1.60 ± 0.33 |  |
| 5-69 | 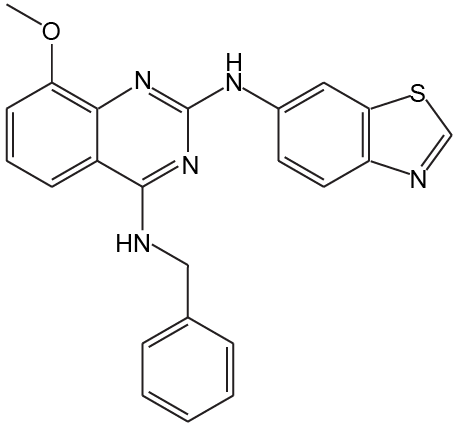 | 51.27 ± 1.54 | 2.10 ± 1.22 |  |
| 5-73 | 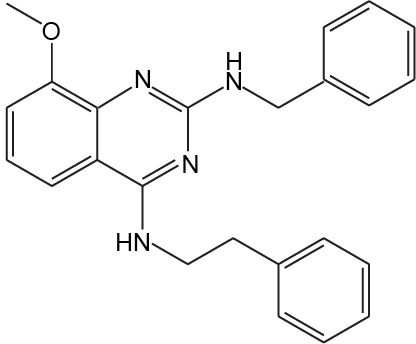 | 41.71 ± 2.35 | 5.34 ± 1.17 |  |
| 5-75 | 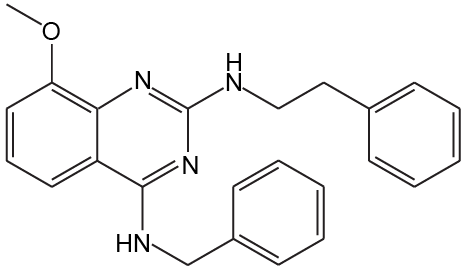 | 45.53 ± 1.40 | 2.25 ± 1.07 |  |
| 5-77-2 (#12) | 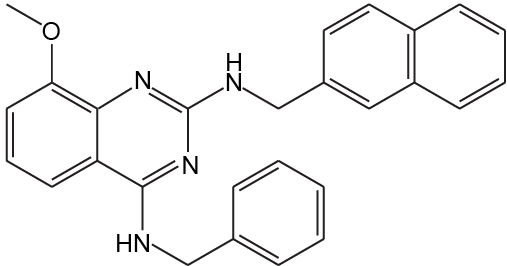 | 30.39 ± 0.43 | 10.42 ± 6.42 |  |
| 5-79 | 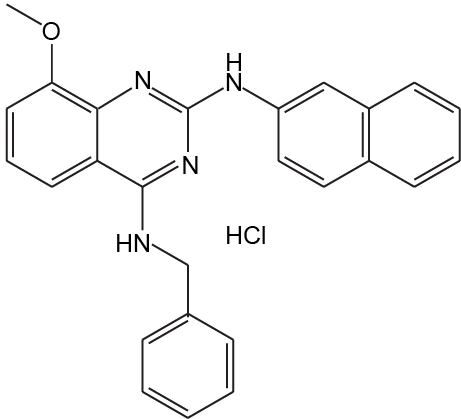 | 76.35 ± 3.47 | 3.40 ± 0.70 |  |
| 5-81 | 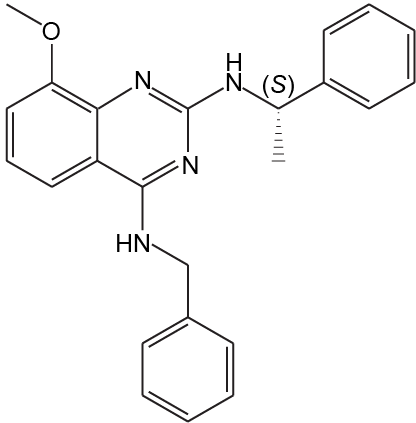 | 43.91 ± 0.05 | 7.65 ± 7.10 |  |
| 5-83 | 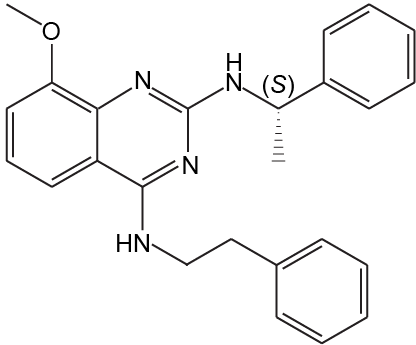 | 42.92 ± 3.93 | 1.83 ± 1.02 |  |
| 5-85 (#13) | 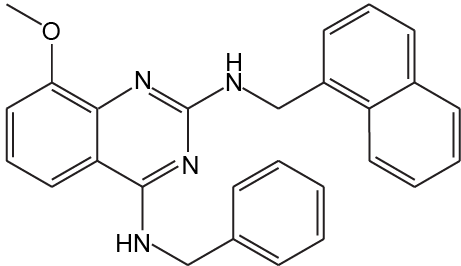 | 28.27 ± 0.04 | 36.79 ± 0.89 | 98.67 ± 2.86 |
| 5-87 (#10) | 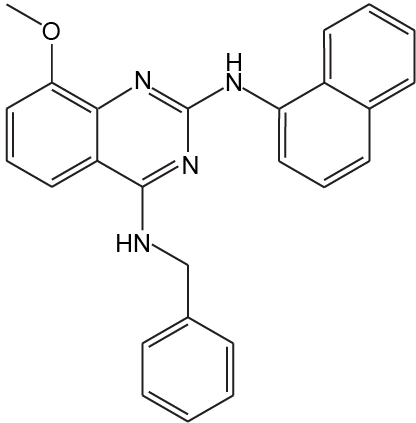 | 49.12 ± 2.89 | 3.97 ± 0.16 |  |
| 5-89 | 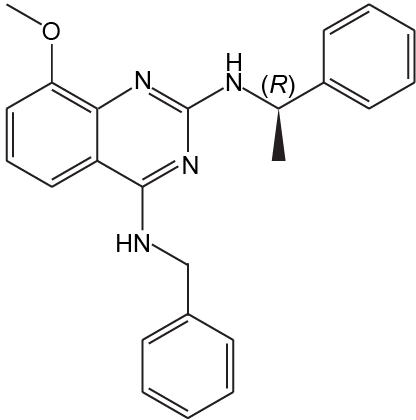 | 46.68 ± 2.80 | 9.13 ± 2.82 |  |
| 5-91 | 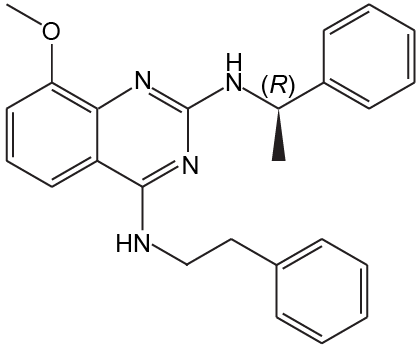 | 40.44 ± 0.01 | 1.69 ± 0.12 |  |
| 5-93 | 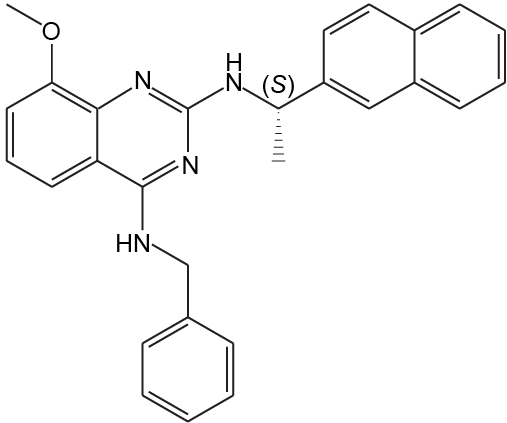 | 58.00 ± 2.01 | 5.34 ± 4.29 |  |
| 5-95 | 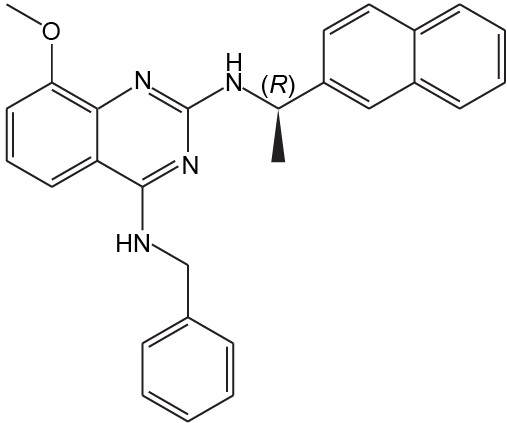 | 42.87 ± 1.98 | 2.43 ± 1.04 |  |
| 5-97 | 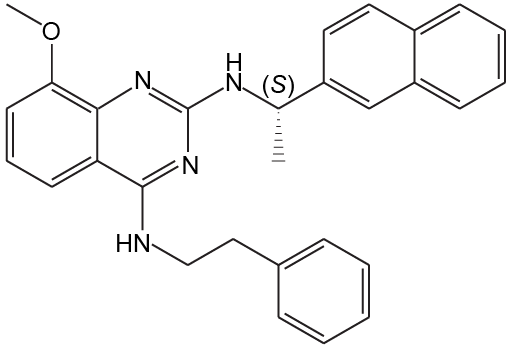 | 49.73 ± 0.04 | 1.82 ± 0.02 |  |
| 5-99 | 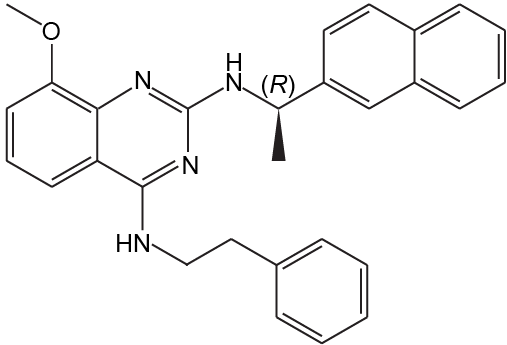 | 45.77 ± 2.14 | 3.40 ± 1.02 |  |
| 5-101 | 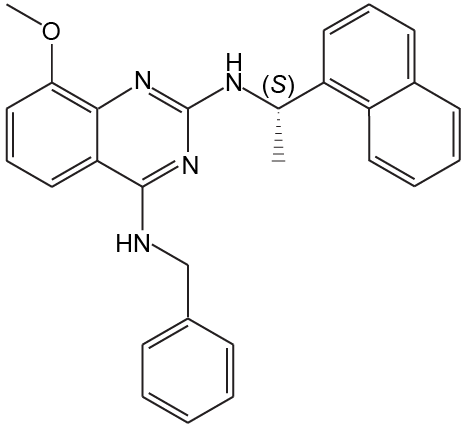 | 49.27 ± 0.19 | 2.43 ± 1.02 |  |
| 5-103 | 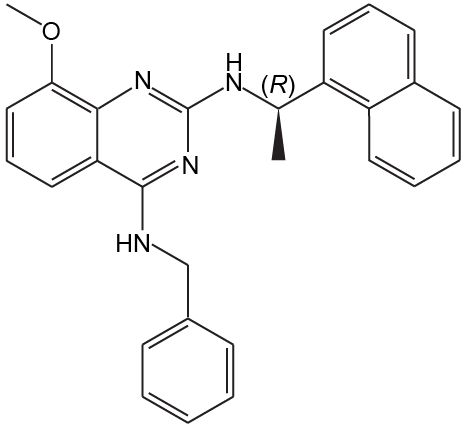 | 45.60 ± 2.79 | 4.36 ± 1.56 |  |
| 5-105 | 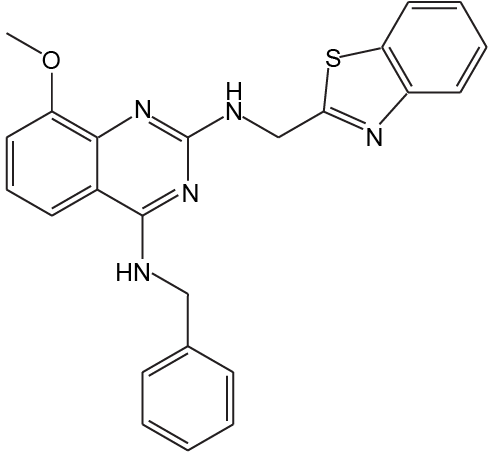 | 56.25 ± 2.23 | 1.30 ± 1.20 |  |
| 5-107 | 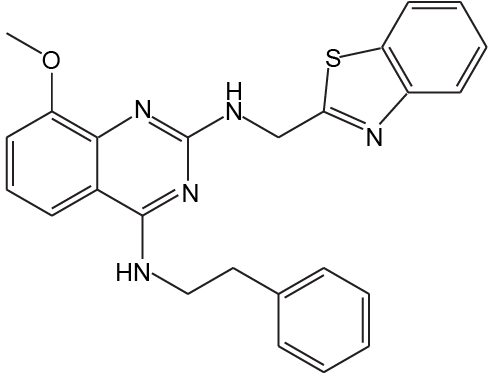 | 75.99 ± 2.63 | 0.24 ± 0.08 |  |
| 5-115 (#2) | 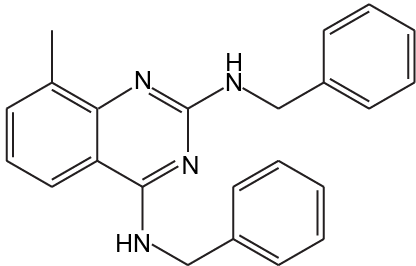 | 55.31 ± 5.04 | 6.69 ± 3.07 |  |
| 5-119 | 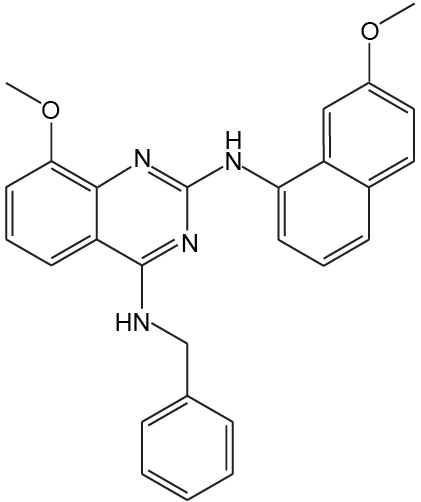 | 53.27 ± 1.65 | 2.49 ± 0.70 |  |
| 5-121 (#11) | 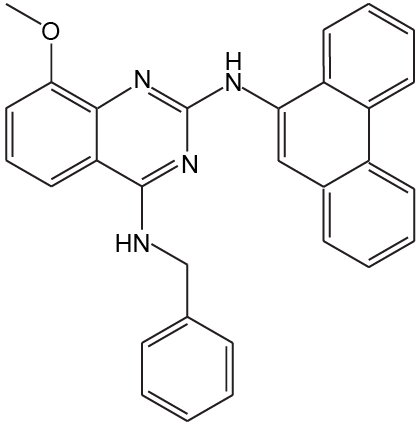 | 64.95 ± 1.17 | 2.05 ± 0.32 |  |
| 5-125 (#3) | 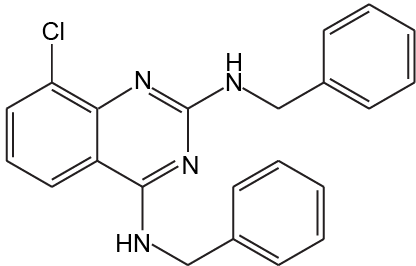 | 71.88 ± 3.41 | 3.86 ± 2.41 |  |
| 5-129 | 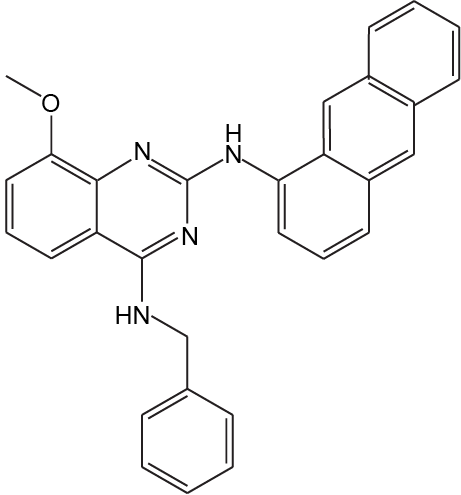 | 63.24 ± 0.19 | 1.25 ± 0.86 |  |
| 5-133 (#4) | 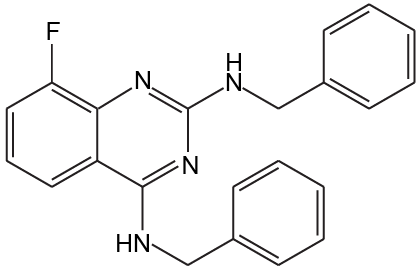 | 75.23 ± 0.63 | 2.92 ± 2.81 |  |
| 5-135 | 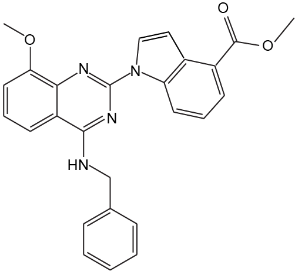 | 86.66 ± 2.70 | 4.63 ± 2.05 |  |
| 5-137 | 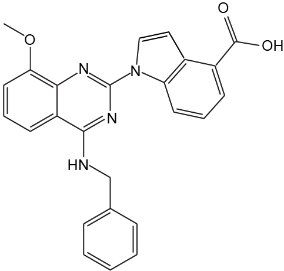 | 96.09 ± 2.11 | 2.21 ± 0.41 |  |
| 5-139 | 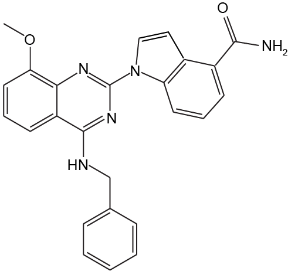 | 90.05 ± 5.58 | 1.75 ± 0.11 |  |
| 5-143 | 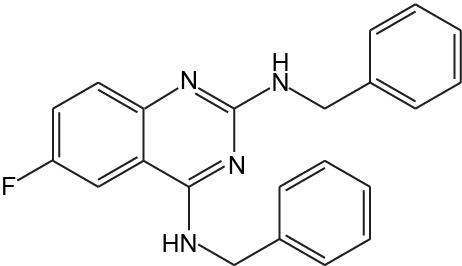 | 48.87 + 0.55 | 11.12 ± 2.40 |  |
| 5-147 (#7) | 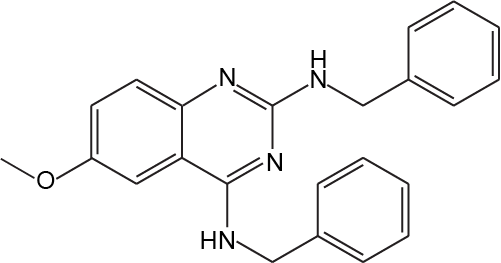 | 43.86 ± 1.20 | 8.92 ± 2.87 |  |
| 5-151 | 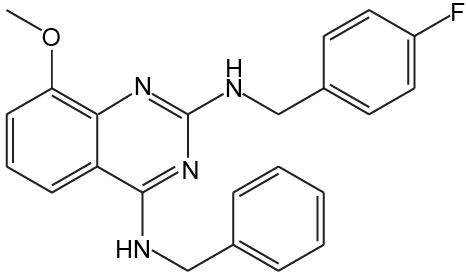 | 47.66 ± 0.34 | 73.02 ± 27.67 | 93.82 ± 0.90 |
| 5-153 (#5) | 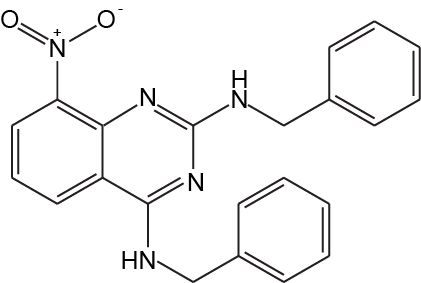 | 86.38 ± 2.37 | 15.05 ± 0.55 |  |
| 5-155 (#8) | 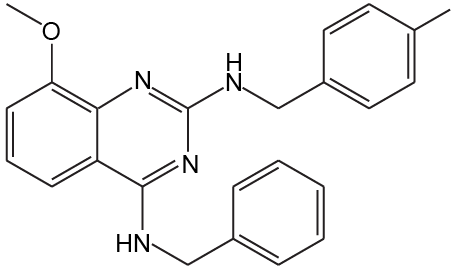 | 27.55 ± 0.01 | 48.09 ± 1.26 | 83.40 ± 3.28 |
| 7-03 (#20) | 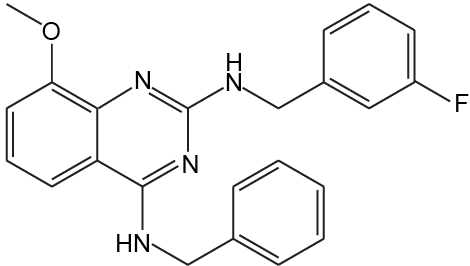 | 35.22 ± 0.66 | 99.48 ± 22.3 | 80.50 ± 0.40 |
| 7-05 | 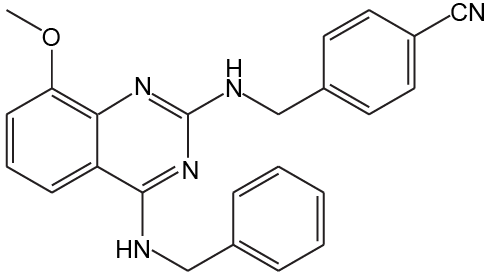 | 54.50 ± 2.02 | 42.59 ± 0.15 | 95.43 ± 0.86 |
| 7-07 (#16) | 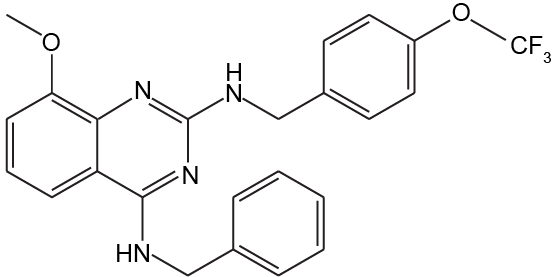 | 41.50 ± 2.98 | 7.52 ± 1.95 |  |
| 7-09 (#15) | 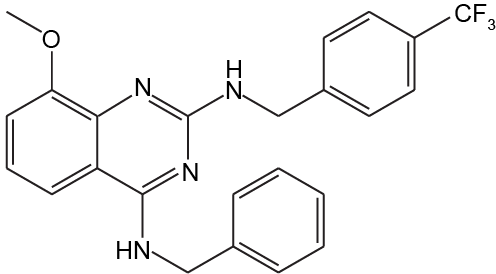 | 28.57 ± 1.13 | 25.61 ± 20.09 |  |
| 7-11 | 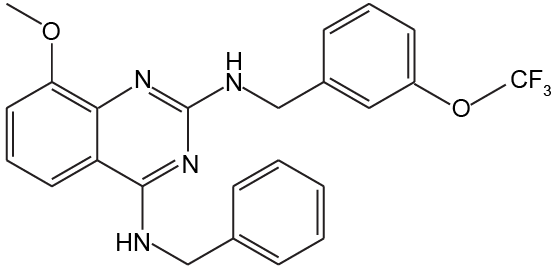 | 46.29 ± 0.61 | 22.15 ± 1.19 |  |
| 7-13 | 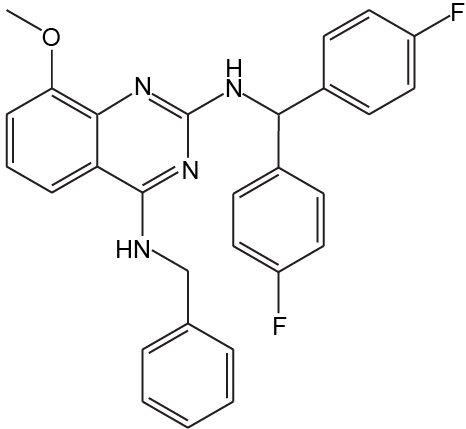 | 39.86 ± 1.36 | 12.60 ± 1.04 |  |
| 7-21 (#6) | 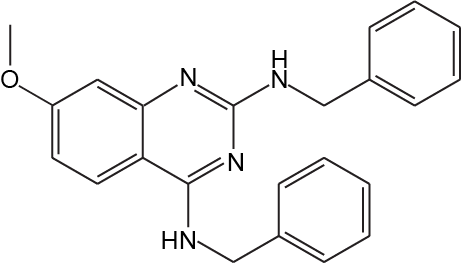 | 35.59 ± 2.28 | 33.47 ± 15.62 | 86.15 ± 0.65 |
| 7-23 | 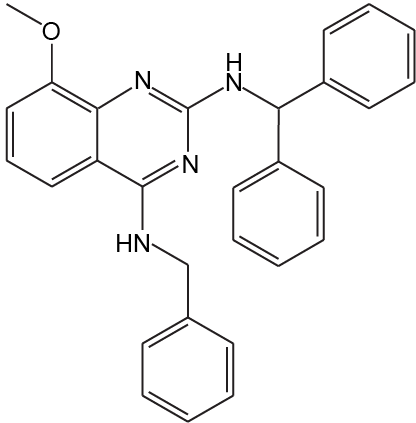 | 37.77 ± 2.37 | 23.20 ± 6.42 |  |
| 7-27 | 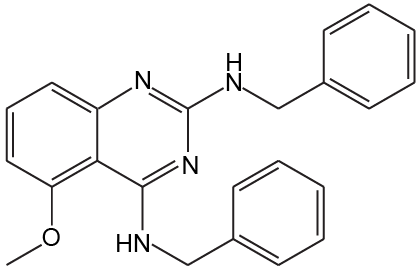 |  | 19.55 ± 2.22 |  |
| 7-31 | 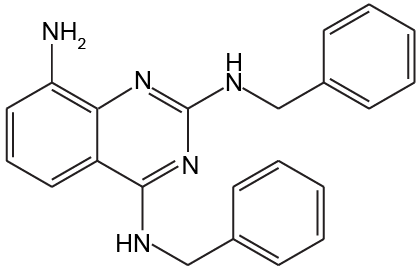 |  | 48.41 ± 2.74 | 69.70 ± 8.05 |
| 7-41 | 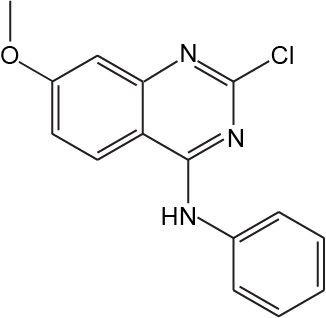 |  | 17.93 ± 2.08 |  |
| 7-45 | 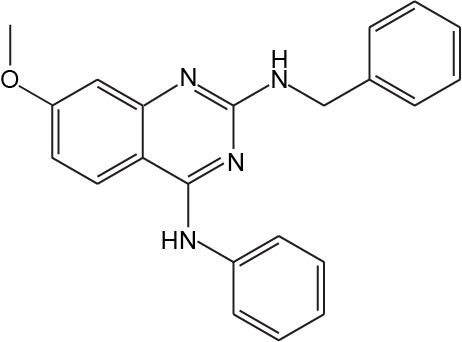 |  | 24.94 ± 5.94 |  |
| 7-65 | 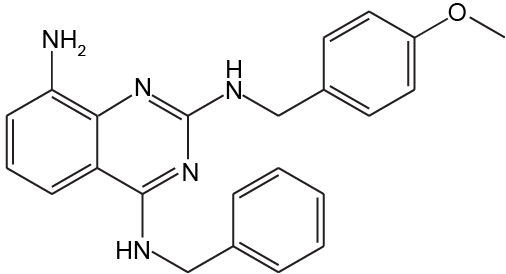 |  | 17.36 ± 5.75 |  |
| 7-69 | 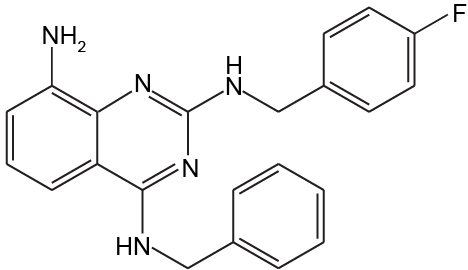 |  | 20.04 ± 6.12 |  |
| 7-75 | 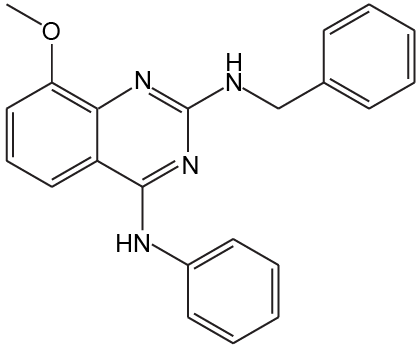 |  | 11.57 ± 12.42 |  |
| 7-77 | 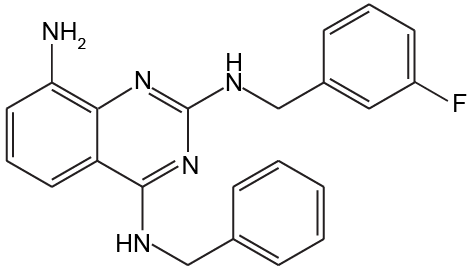 |  | 26.24 ± 20.18 |  |

^a, c^ Screened at 5 µM. Data are mean ± S.D. from two independent experiments, each with technical triplicates. ^b^ Screened at 1 µM. Data are mean ± S.D. from at least one screen, each with technical triplicates. * Purchased. (#) Compound identifier used in main text. Blank cells indicate compound was not tested in that assay.
